# Association between sleep behaviors and adiposity indices among U.S. adults: a cross-sectional study

**DOI:** 10.3389/fnut.2025.1526422

**Published:** 2025-03-14

**Authors:** Shenghao Xu, Jie Lin, Qibo Xu, Kai Zhao, Jianlin Xiao

**Affiliations:** ^1^Department of Orthopedics, China-Japan Union Hospital of Jilin University, Changchun, China; ^2^Department of Orthopedics, The Second Hospital of Jilin University, Changchun, China; ^3^Department of Hepatobiliary and Pancreatic Surgery, The Second Hospital of Jilin University, Changchun, China

**Keywords:** sleep duration, sleep disorder, adiposity indices, cross-sectional study, NHANES

## Abstract

**Objectives:**

This study aimed to clarify the relationship between sleep behaviors and adiposity indices.

**Methods:**

We analyzed NHANES data from 2011 to 2018 for adults aged 20–80, assessing BMI, waist circumference (WC), lean mass, and body fat percentage with DEXA scans and physical measurements. Sleep duration was categorized into short (<7 h), normal (7–9 h), and long (>9 h), as well as their sleep status based on questionnaires. Furthermore, we examined the interaction effects between sleep duration and sleep patterns.

**Results:**

Among 19,951 participants providing BMI and WC data, and 10,716 for lean mass and body fat percentage, short sleep duration correlated with higher BMI (β = 0.56, 95% CI: 0.36–0.76), WC (β = 0.90, 95% CI: 0.43–1.37), and lean mass (β = 0.70, 95% CI: 0.32–1.07). Individuals with sleep disorders showed increased values across all indices: BMI (β = 0.93, 95% CI: 0.72–1.13), WC (β = 2.40, 95% CI: 1.92–2.88), lean mass (β = 0.71, 95% CI: 0.30–1.12), and body fat percentage (β = 0.64, 95% CI: 0.37–0.90). No significant interaction effects were found between sleep duration and sleep disorders.

**Conclusion:**

Our findings indicate that individuals with short sleep duration and sleep disorders are likely to carry a higher weight burden, indicating potential targets for addressing obesity-related health issues.

## Introduction

Sleep is a crucial component of overall health, affecting both physical and mental well-being. The Sleep Research Society and the American Academy of Sleep Medicine recommend at least 7 h of sleep per night for optimal health in adults (1, 2). However, modern lifestyle factors, including increased use of electronic devices and rising social pressures, have significantly reduced average sleep duration from 9 h per night in the early 20th century to less than 7 h today (3). Insufficient sleep adversely impacts cognitive functions, learning, memory, work efficiency, and social skills (4). Furthermore, it increases the risk of various health issues such as obesity, hypertension, cardiovascular diseases (5–8) and even premature death (9, 10). Sleep disorders often co-occur with insufficient sleep, presenting a significant global challenge. A survey study in the United States revealed that approximately 60 million Americans suffer from sleep disorders (11, 12). The combination of these issues exacerbates the burden of various global health problems, particularly obesity.

The interplay between sleep and obesity has garnered considerable attention. Studies have shown that sleeping ≤6 h per night is associated with a higher body mass index (BMI) (13, 14). Adequate sleep and good sleep quality have been shown to promote weight loss (15), suggesting that sleep is a significant factor in weight management. Epidemiological and experimental studies consistently demonstrate that short sleep duration is a notable risk factor for weight gain and obesity (16), leading to various health problems (17, 18). While most studies have focused on the relationship between sleep and BMI or obesity, fewer have explored other adiposity indices such as waist circumference (WC), lean body mass, and body fat percentage. These measures provide a more comprehensive understanding of body composition and its relationship with sleep behaviors. Therefore, it is essential to investigate these associations using a representative sample to identify potential public health interventions.

This study aims to address this gap by examining the relationships between various sleep behaviors and multiple adiposity indices—BMI, WC, lean body mass, and body fat percentage—using data from the National Health and Nutrition Examination Survey (NHANES). By analyzing a large, nationally representative sample of U.S. adults, this study seeks to provide new insights into how sleep behaviors influence body composition, thereby informing preventive strategies for obesity.

## Methods

### Study design and population

This cross-sectional study utilized data from the NHANES conducted between 2011 and 2018. The NHANES is a program managed by the National Center for Health Statistics (NCHS) of the Centers for Disease Control and Prevention (CDC), which aims to assess the health and nutritional status of adults and children in the United States. NHANES provides a large, nationally representative sample of U.S. adults, employs a rigorous multistage probability sampling design, and includes comprehensive anthropometric and sleep-related data collected through standardized protocols. These features make NHANES particularly well-suited for examining the associations between sleep behaviors and adiposity indices in a general population.

**Figure 1:**
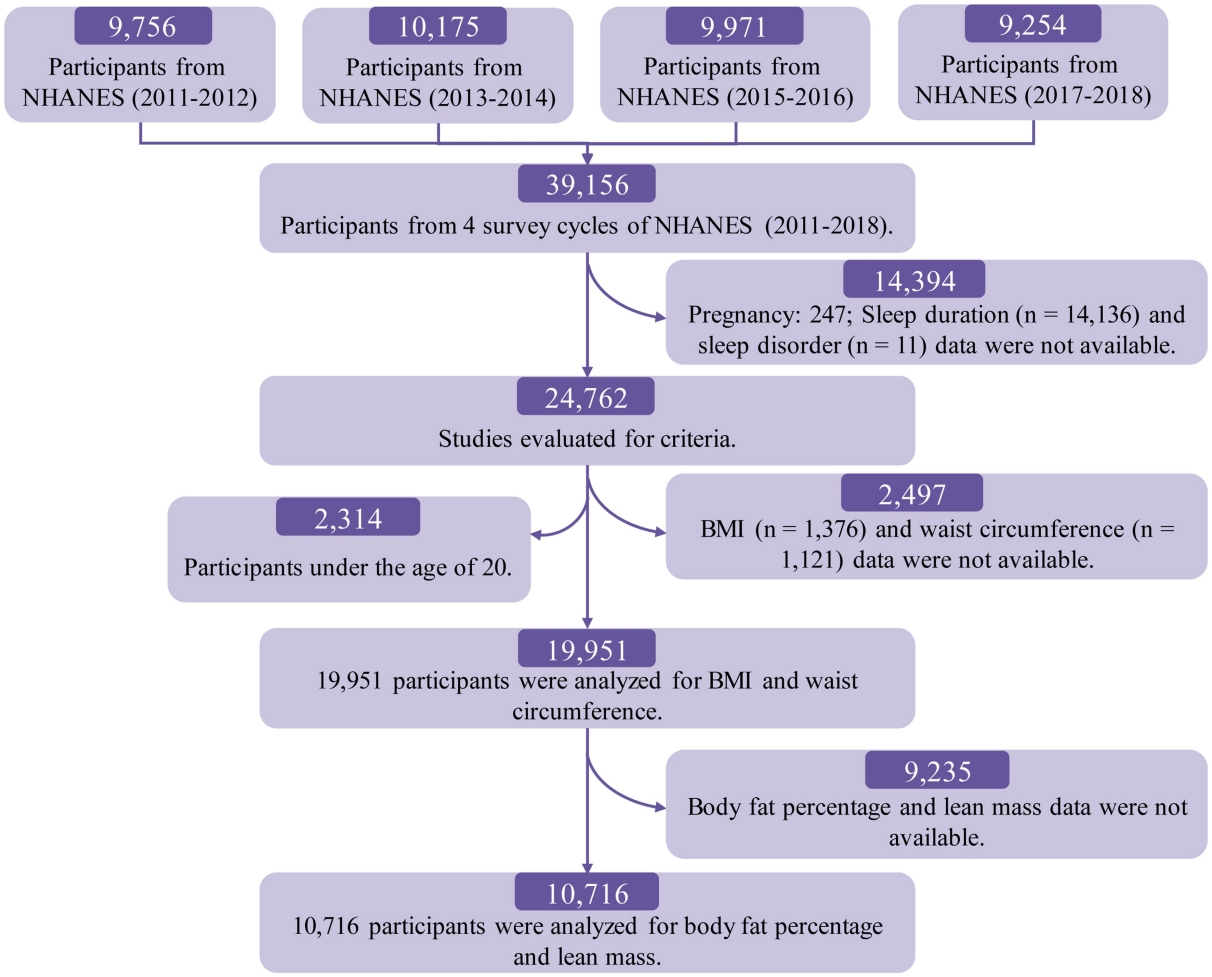
Flowchart showing study participant selection.

For this study, data from four consecutive NHANES cycles (2011–2018) were used, encompassing a total of 39,156 participants. Participants were excluded if they were under 20 years old, pregnant, or had missing data on key variables. After applying these exclusion criteria, the final analytical sample included 19,951 participants for BMI and waist circumference analysis and 10,716 participants for lean body mass and body fat percentage analysis. The detailed participant selection process is shown in Figure 1.

All NHANES protocols were approved by the NCHS Research Ethics Review Board, and informed consent was obtained from all participants. Detailed information on the survey design, participant recruitment, and data collection procedures is available on the CDC NHANES website.^1^

### Sleep duration and sleep disorder

Sleep duration was assessed through a questionnaire that asked participants, “How many hours do you usually sleep on weekdays or workdays?” Responses were recorded in half-hour increments and treated as a continuous variable. For analysis, sleep duration was categorized into three groups: short sleep (<7 h), normal sleep (7–9 h), and long sleep (>9 h) (19, 20).

To assess sleep disorders, participants responded to the NHANES household interview survey’s “Sleep Disorders” questionnaire. The questions included: “Ever told doctor had trouble sleeping?” or “Ever told by doctor have sleep disorder?” Participants who answered “yes” to either question were classified as having a sleep disorder, while those who answered “no” to both were considered not to have sleep problems. This method allowed for a comprehensive evaluation of both sleep duration and sleep disorders among the study participants, facilitating an in-depth analysis of their potential associations with various adiposity indices.

### Body measures

Participants were weighed at the mobile examination center, with weights recorded in kilograms using a digital scale. Height was measured using a stadiometer, and waist circumference was measured with a steel tape above the iliac crest. Detailed measurement protocols, equipment, and quality control procedures are described in the NHANES Anthropometry Procedures Manual (21). BMI was calculated as weight in kilograms divided by height in meters squared.

Body composition, including lean body mass and body fat percentage, was measured using dual-energy X-ray absorptiometry by trained and certified radiologic technologists. The measurements were conducted on adults aged 20–59 years using a Hologic QDR 4500A fan-beam densitometer. Data was analyzed using Hologic APEX version 4.0 software with the NHANES Body Composition Analysis option.

### Covariates

This study collected and categorized various health data from participants. Hypertension was defined as a systolic blood pressure of ≥140 mmHg and/or diastolic blood pressure of ≥90 mmHg, self-reported hypertension, or the use of antihypertensive medications (22). Diabetes was diagnosed based on a physician’s diagnosis, glycated hemoglobin (HbA1c) levels >6.5%, or the use of diabetes medications or insulin (22). Depression was assessed using the Patient Health Questionnaire-9, with a score of ≥10 indicating depression (23). Other self-reported medical conditions included stroke, thyroid disease, and cancer. Alcohol consumption was classified as more than two drinks per day for men and more than one drink per day for women. Smoking status was determined by whether participants had smoked at least 100 cigarettes in their lifetime, and they were categorized as current smokers (smoked more than 100 cigarettes in life and smoke some days or every day), former smokers (smoked more than 100 cigarettes in life, not at all), or never smokers (smoked less than 100 cigarettes in life) (24). Energy intake was calculated from the average of two 24-h dietary recall interviews. Physical activity was quantified using the overall metabolic equivalent (MET) score for various activities, and sedentary time was recorded as the time spent sitting during a typical day, excluding sleep. Comprehensive data from this study is accessible on the NHANES website, which provides additional details on the survey design and data collection procedures.

### Statistical analysis

The analysis was conducted using weighted data according to NHANES 2011–2018 guidelines, weighted by using 1/4 * wtmec2yr. Continuous variables are presented as means and standard deviations (SD) or median (inter-quartile range), while categorical variables are shown as percentages. Depending on whether the continuous variables were normally or skewed distributed, the choice was made to use either the one-way ANOVA or the Kruskal-Wallis H-test. For categorical variables, the chi-square test was used. Effect sizes are expressed as β values with 95% confidence intervals (CIs).

To examine the relationships between sleep duration and patterns with BMI, waist circumference, lean body mass, and body fat percentage, multivariate regression models were used. Three models were constructed: Model 1 was unadjusted, Model 2 adjusted for gender, age, and race, and Model 3 adjusted for all covariates, including education level, marital status, poverty-income ratio, alcohol consumption, smoking, energy intake, hypertension, diabetes, stroke, depression, thyroid disease, and cancer.

A gender-stratified smoothing curve fitting method was employed to analyze the relationship between sleep duration and the four adiposity indices, with the log-likelihood ratio test used to assess inflection points. Stratified analyses were also performed to evaluate the impact of sleep duration and patterns on the adiposity indices across different populations. Interaction tests between sleep duration and sleep patterns were conducted. *P* value of <0.05 was considered statistically significant. All analyses were performed using R^2^ and EmpowerStats^3^ (X&Y Solutions Inc.).

## Results

In this study, we used different sample sizes for various outcome variables: 19,951 for BMI and WC, and 10,716 for lean body mass and body fat percentage. Table 1 presents the baseline characteristics categorized by sleep behaviors, showing that all included variables differed significantly among the three groups. Supplementary Table S1 shows the weighted baseline characteristics. The proportion of older adults and females was higher in the long sleep group. Participants in the normal and short sleep groups engaged in more physical activity, had shorter sedentary time, and had higher energy intake. In addition, participants with sleep disorders were primarily older adults and females, had a higher comorbidity index, spent longer sedentary time, and were more likely to smoke. Additionally, participants with sleep disorders had a higher BMI, WC, lean body mass, and body fat percentage.

**Table 1:** Characteristics of participants by categories of weekday sleep duration and sleep pattern status: NHANES 2011–2018.

	<7 h (*n* = 6,353)	7–9 h (*n* = 12,227)	>9 h (*n* = 1,371)	*P* value	Healthy (*n* = 14,508)	Sleep disorder (*n* = 5,443)	*P* value
Age (years, median [IQR])	48.00 [35.00, 61.00]	49.00 [34.00, 64.00]	56.00 [33.00, 70.00]	<0.001	47.00 [33.00, 62.00]	54.00 [41.00, 65.00]	<0.001
Physical activity (MET-h, median [IQR])	12.00 [4.00, 28.00]	10.00 [4.00, 24.00]	8.70 [4.00, 24.00]	<0.001	12.00 [4.00, 25.30]	9.70 [4.00, 23.00]	<0.001
Sedentary time (hours, median [IQR])	6.00 [4.00, 8.00]	6.00 [4.00, 8.00]	5.00 [3.00, 8.00]	0.061	5.00 [3.00, 8.00]	6.00 [4.00, 8.00]	<0.001
Energy intake (kcal/d, median [IQR])	1934.50 [1469.50, 2528.87]	1921.50 [1479.00, 2478.50]	1759.00 [1324.25, 2316.38]	<0.001	1932.50 [1485.00, 2505.50]	1872.50 [1424.12, 2427.75]	
Weight (kg, median [IQR])	80.50 [68.00, 96.20]	77.50 [65.60, 91.70]	76.70 [64.97, 91.85]	<0.001	77.20 [65.50, 91.10]	82.40 [68.80, 98.82]	<0.001
BMI (kg/m^2^, median [IQR])	28.60 [24.70, 33.60]	27.80 [24.10, 32.40]	28.30 [24.27, 33.00]	<0.001	27.60 [24.00, 32.00]	29.65 [25.30, 34.70]	<0.001
Waist (cm, median [IQR])	99.00 [88.50, 110.90]	97.50 [87.10, 108.30]	99.00 [87.97, 111.32]	<0.001	96.80 [86.60, 107.30]	102.40 [91.20, 114.70]	<0.001
Lean mass (kg, median [IQR])	55.20 [45.40, 64.60]	52.40 [43.20, 61.80]	50.35 [41.65, 60.70]	<0.001	53.10 [43.70, 62.30]	53.80 [44.55, 64.30]	<0.001
Body fat percentage (%, median [IQR])	32.50 [26.30, 39.70]	32.80 [26.70, 39.80]	34.35 [26.42, 41.00]	0.166	31.80 [26.00, 39.20]	35.30 [28.70, 41.60]	<0.001
Gender (%)				<0.001			<0.001
Male	3299.00 (51.93)	5930.00 (48.50)	596.00 (43.47)		7491.00 (51.63)	2334.00 (42.88)	
Female	3054.00 (48.07)	6297.00 (51.50)	775.00 (56.53)		7017.00 (48.37)	3109.00 (57.12)	
Race (%)				<0.001			<0.001
Mexican American	766.00 (12.06)	1731.00 (14.16)	211.00 (15.39)		2155.00 (14.85)	553.00 (10.16)	
Other Hispanic	702.00 (11.05)	1253.00 (10.25)	143.00 (10.43)		1575.00 (10.86)	523.00 (9.61)	
Non-Hispanic White	2006.00 (31.58)	4829.00 (39.49)	520.00 (37.93)		4812.00 (33.17)	2543.00 (46.72)	
Non-Hispanic Black	1904.00 (29.97)	2287.00 (18.70)	316.00 (23.05)		3313.00 (22.84)	1194.00 (21.94)	
Non-Hispanic Asian	728.00 (11.46)	1717.00 (14.04)	129.00 (9.41)		2196.00 (15.14)	378.00 (6.94)	
Other Race	247.00 (3.89)	410.00 (3.35)	52.00 (3.79)		457.00 (3.15)	252.00 (4.63)	
Education level (%)				< 0.001			<0.001
Less than 9th grade	523.00 (8.23)	1105.00 (9.04)	184.00 (13.42)		1420.00 (9.79)	392.00 (7.20)	
9–11th grade	843.00 (13.27)	1402.00 (11.47)	269.00 (19.62)		1843.00 (12.70)	671.00 (12.33)	
High school graduate	1468.00 (23.11)	2618.00 (21.41)	349.00 (25.46)		3188.00 (21.97)	1247.00 (22.91)	
Some college or AA degree	2124.00 (33.43)	3658.00 (29.92)	369.00 (26.91)		4281.00 (29.51)	1870.00 (34.36)	
College graduate or above	1393.00 (21.93)	3435.00 (28.09)	198.00 (14.44)		3765.00 (25.95)	1261.00 (23.17)	
Missing	2.00 (0.03)	9.00 (0.07)	2.00 (0.15)		11.00 (0.08)	2.00 (0.04)	
Marital status (%)				<0.001			<0.001
Married	3084.00 (48.54)	6482.00 (53.01)	541.00 (39.46)		7556.00 (52.08)	2551.00 (46.87)	
Widowed	400.00 (6.30)	851.00 (6.96)	152.00 (11.09)		913.00 (6.29)	490.00 (9.00)	
Divorced	798.00 (12.56)	1234.00 (10.09)	149.00 (10.87)		1356.00 (9.35)	825.00 (15.16)	
Separated	281.00 (4.42)	350.00 (2.86)	58.00 (4.23)		454.00 (3.13)	235.00 (4.32)	
Never married	1253.00 (19.72)	2286.00 (18.70)	319.00 (23.27)		2943.00 (20.29)	915.00 (16.81)	
Living with partner	533.00 (8.39)	1021.00 (8.35)	150.00 (10.94)		1279.00 (8.82)	425.00 (7.81)	
Missing	4.00 (0.06)	3.00 (0.02)	2.00 (0.15)		7.00 (0.05)	2.00 (0.04)	
Poverty income ratio (%)				<0.001			<0.001
≤1.3	1960.00 (30.85)	3341.00 (27.32)	566.00 (41.28)		4160.00 (28.67)	1707.00 (31.36)	
>1.3 and ≤3.5	2151.00 (33.86)	4126.00 (33.74)	468.00 (34.14)		4926.00 (33.95)	1819.00 (33.42)	
>3.5	1653.00 (26.02)	3622.00 (29.62)	196.00 (14.30)		3999.00 (27.56)	1472.00 (27.04)	
Missing	589.00 (9.27)	1138.00 (9.31)	141.00 (10.28)		1423.00 (9.81)	445.00 (8.18)	
Alcohol (%)				<0.001			0.274
No	1961.00 (30.87)	4029.00 (32.95)	371.00 (27.06)		4601.00 (31.71)	1760.00 (32.34)	
Yes	2096.00 (32.99)	3794.00 (31.03)	392.00 (28.59)		4544.00 (31.32)	1738.00 (31.93)	
Missing	2296.00 (36.14)	4404.00 (36.02)	608.00 (44.35)		5363.00 (36.97)	1945.00 (35.73)	
Smoking (%)				<0.001			<0.001
Non-users	3433.00 (54.04)	7238.00 (59.20)	743.00 (54.19)		8832.00 (60.88)	2582.00 (47.44)	
Current smoking	1539.00 (24.22)	2065.00 (16.89)	308.00 (22.47)		2599.00 (17.91)	1313.00 (24.12)	
Past smoking	1377.00 (21.67)	915.00 (23.84)	318.00 (23.19)		3065.00 (21.13)	1545.00 (28.39)	
Missing	4.00 (0.06)	9.00 (0.07)	2.00 (0.15)		12.00 (0.08)	3.00 (0.06)	
Hypertension (%)				<0.001			<0.001
No	3460.00 (54.46)	7225.00 (59.09)	674.00 (49.16)		8971.00 (61.83)	2388.00 (43.87)	
Yes	2887.00 (45.44)	4993.00 (40.84)	695.00 (50.69)		5526.00 (38.09)	3049.00 (56.02)	
Missing	6.00 (0.09)	9.00 (0.07)	2.00 (0.15)		11.00 (0.08)	6.00 (0.11)	
Diabetes (%)				<0.001			<0.001
No	5094.00 (80.18)	10118.00 (82.75)	1022.00 (74.54)		12204.00 (84.12)	4030.00 (74.04)	
Yes	1117.00 (17.58)	1903.00 (15.56)	328.00 (23.92)		2076.00 (14.31)	1272.00 (23.37)	
Borderline	142.00 (2.24)	206.00 (1.68)	21.00 (1.53)		228.00 (1.57)	141.00 (2.59)	
Depression (%)				<0.001			<0.001
No	5058.00 (79.62)	10424.00 (85.25)	1039.00 (75.78)		12547.00 (86.48)	3974.00 (73.01)	
Yes	774.00 (12.18)	866.00 (7.08)	209.00 (15.24)		705.00 (4.86)	1144.00 (21.02)	
Missing	521.00 (8.20)	937.00 (7.66)	123.00 (8.97)		1256.00 (8.66)	325.00 (5.97)	
Stroke (%)				<0.001			<0.001
No	6115.00 (96.25)	11826.00 (96.72)	1278.00 (93.22)		14091.00 (97.13)	5128.00 (94.21)	
Yes	232.00 (3.65)	391.00 (3.20)	92.00 (6.71)		408.00 (2.81)	307.00 (5.64)	
Missing	6.00 (0.09)	10.00 (0.08)	1.00 (0.07)		9.00 (0.06)	8.00 (0.15)	
Thyroid disease (%)				0.057			<0.001
No	5707.00 (89.83)	10924.00 (89.34)	1204.00 (87.82)		13285.00 (91.57)	4550.00 (83.59)	
Yes	628.00 (9.89)	1280.00 (10.47)	166.00 (12.11)		1204.00 (8.30)	870.00 (15.98)	
Missing	18.00 (0.28)	23.00 (0.19)	1.00 (0.07)		19.00 (0.13)	23.00 (0.42)	
Cancer or malignancy (%)				<0.001			<0.001
No	5831.00 (91.78)	11048.00 (90.36)	1207.00 (88.04)		13377.00 (92.20)	4709.00 (86.51)	
Yes	516.00 (8.12)	1174.00 (9.60)	164.00 (11.96)		1123.00 (7.74)	731.00 (13.43)	
Missing	6.00 (0.09)	5.00 (0.04)	0.00 (0.00)		8.00 (0.06)	3.00 (0.06)	

IQR, interquartile range; kg, kilogram; cm, centimeter; n, number; BMI, body mass index.

### Body mass index

**Figure 2:**
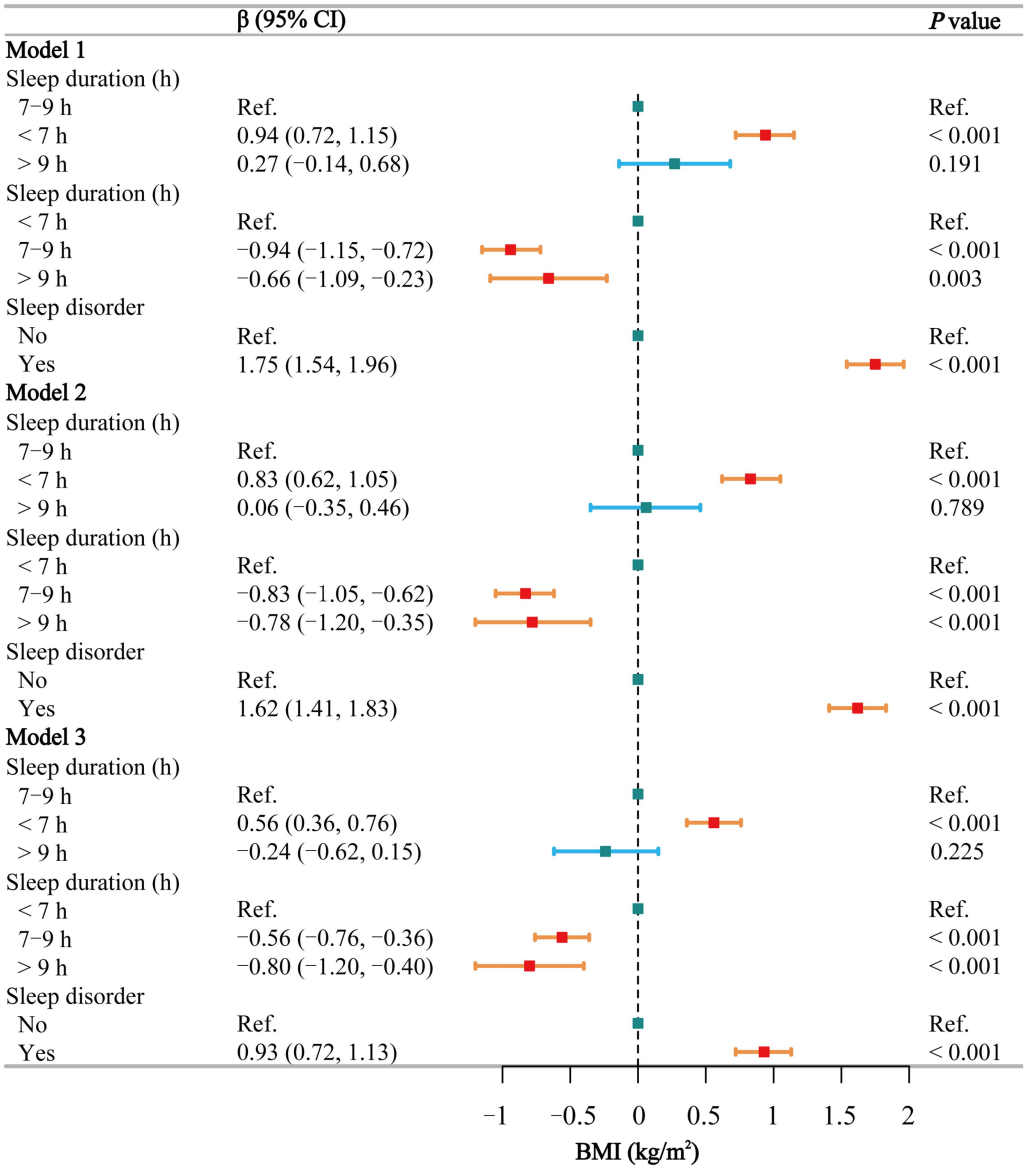
Associations between sleep behaviors and BMI. Model 1 was unadjusted, Model 2 adjusted for gender, age, and race, and Model 3 adjusted for all covariates, including education level, marital status, poverty-income ratio, alcohol consumption, smoking, energy intake, hypertension, diabetes, stroke, depression, thyroid disease, and cancer. CI, confidence interval; h, hour; Ref, reference; BMI, body mass index. *p* < 0.05 was considered statistically significant.

The results of the multivariate regression analysis (Figure 2) indicate that, using normal sleep as a reference, short sleep is positively associated with an increase in BMI in Model 1 (β = 0.94, 95% CI: 0.72 to 1.15), Model 2 (β = 0.83, 95% CI: 0.62 to 1.05), and Model 3 (β = 0.56, 95% CI: 0.36 to 0.76). Long sleep, however, is not associated with BMI. Using short sleep as a reference, BMI is significantly lower in individuals with normal and long sleep durations across all models: Model 1 (β = −0.94 and −0.66, respectively), Model 2 (β = −0.83 and −0.78, respectively), and Model 3 (β = −0.56 and −0.80, respectively). Additionally, individuals with sleep disorders had significantly higher BMI compared to the healthy population in all three models (β = 1.75, 1.62, and 0.93, respectively). Gender-stratified smoothing curve fitting (Supplementary Figure S1A) and threshold effect analyses (Supplementary Table S2) revealed a negative linear relationship between sleep duration and BMI in males (β = −0.13, *p* = 0.002). In females, a segmented effect was observed: within the 5–12 h range, sleep duration was negatively correlated with BMI (β = −0.29, *p* < 0.001), while beyond 12 h, sleep duration was positively correlated with BMI (β = 10.77, *p* < 0.001).

In the subgroup analysis of the relationship between sleep duration and BMI (Supplementary Table S3), short sleep was positively associated with BMI among non-smokers, non-drinkers, individuals without diseases, those with higher energy intake, and females. The stratified logistic regression analysis of sleep disorders (Supplementary Table S4) indicated that nearly all individuals with sleep disorders had a higher BMI. The interaction test (Supplementary Table S5) between sleep duration and sleep disorders did not reveal any significant interaction (*p* = 0.215).

### Waist circumference

**Figure 3:**
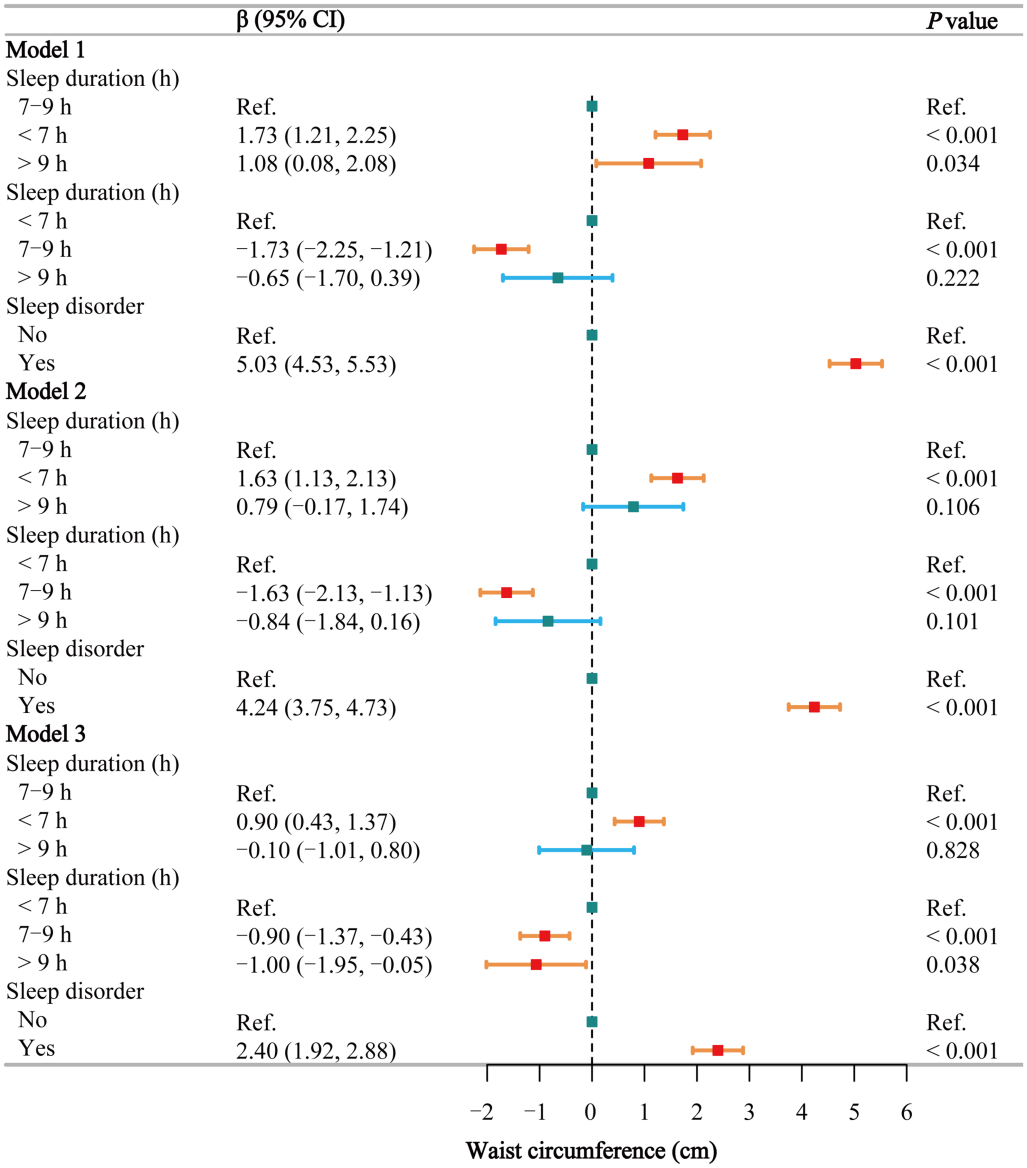
Associations between sleep behaviors and waist circumference. Model 1 was unadjusted, Model 2 adjusted for gender, age, and race, and Model 3 adjusted for all covariates, including education level, marital status, poverty-income ratio, alcohol consumption, smoking, energy intake, hypertension, diabetes, stroke, depression, thyroid disease, and cancer. CI, confidence interval; h, hour; Ref, reference. *p* < 0.05 was considered statistically significant.

The multivariate regression analysis (Figure 3) shows that, using normal sleep duration as a reference, short sleep is positively associated with an increase in WC in the fully adjusted Model 3 (β = 0.90, 95% CI: 0.43–1.37), while long sleep shows no such association. Compared to short sleep, participants with normal and long sleep had lower WC across all three models, but the differences were significant only in Model 3 (β = −0.90 and −1.00, respectively). In all models, individuals with sleep disorders had significantly higher WC (β = 5.03, 4.24, and 2.40, respectively). Smoothing curve fitting (Supplementary Figure S1B) and threshold effect analyses (Supplementary Table S6) show a negative linear relationship between sleep duration and WC in males (β = −0.20, *p* = 0.050). In females, a segmented effect was observed (*P* for log-likelihood = 0.040): sleep duration ≤8 h was negatively correlated with WC (β = −0.55, *p* < 0.001), while sleep duration >8 h was positively correlated with WC (β = 0.13, *p* = 0.592), although this was not statistically significant.

In the subgroup analysis of the relationship between sleep duration (Supplementary Table S7) and sleep disorders (Supplementary Table S8) and WC, short sleep and sleep disorders exhibited trends consistent with those observed for BMI. The interaction test (Supplementary Table S9) between sleep duration and sleep disorders did not reveal any significant interaction (*p* = 0.265).

### Lean mass

**Figure 4:**
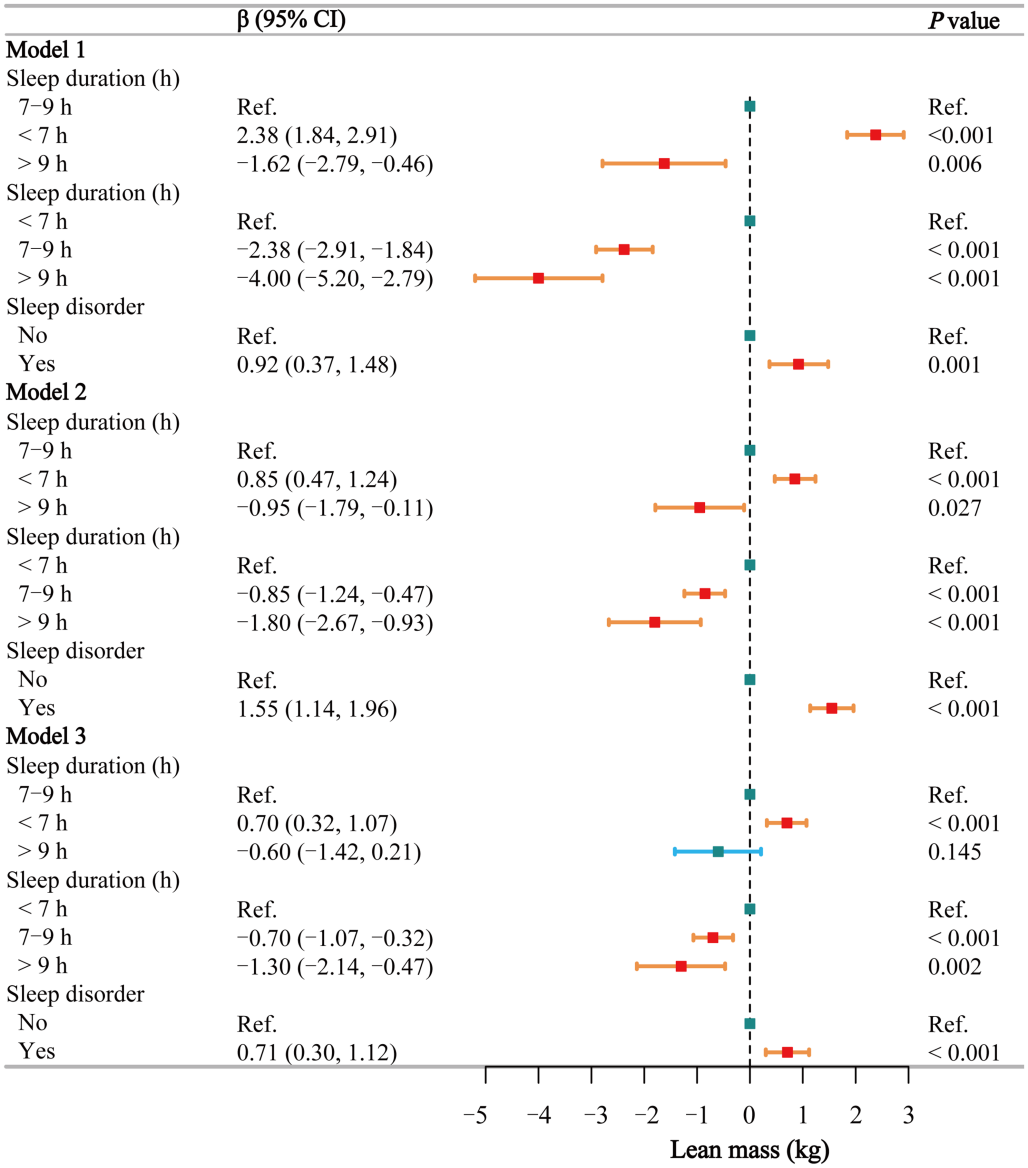
Associations between sleep behaviors and lean mass. Model 1 was unadjusted, Model 2 adjusted for gender, age, and race, and Model 3 adjusted for all covariates, including education level, marital status, poverty-income ratio, alcohol consumption, smoking, energy intake, hypertension, diabetes, stroke, depression, thyroid disease, and cancer. CI, confidence interval; h, hour; Ref, reference. *p* < 0.05 was considered statistically significant.

The multivariate regression analysis (Figure 4) shows that in Model 3, short sleep is positively associated with an increase in lean body mass (*β* = 0.70, 95% CI: 0.32–1.07), while normal sleep (β = −0.70, 95% CI: −1.07 to −0.32) and long sleep (β = −1.30, 95% CI: −2.14 to −0.47) are negatively associated with lean body mass. Similarly, in all three models, individuals with sleep disorders had significantly higher lean body mass compared to the healthy population (β = 0.92, 1.55, and 0.71, respectively). Smoothing curve fitting (Supplementary Figure S1C) and threshold effect analyses (Supplementary Table S10) indicate a negative linear relationship between sleep duration and lean body mass in both males and females (*P* for log-likelihood >0.05), suggesting that lean body mass decreases with increasing sleep duration, and these trends are statistically significant (*p* = 0.001 and 0.020, respectively).

In the subgroup analysis of the relationship between sleep duration and lean body mass (Supplementary Table S11), short sleep was positively associated with lean body mass in both males and females, particularly among those without underlying diseases. Unlike BMI and waist circumference, the impact of long sleep on lean body mass was limited. The stratified logistic regression analysis of sleep disorders (Supplementary Table S12) showed that the effect of sleep disorders on lean body mass was smaller compared to their impact on BMI and waist circumference. The interaction test (Supplementary Table S13) between sleep duration and sleep disorders did not reveal any significant interaction (*p* = 0.402).

### Body fat percentage

**Figure 5:**
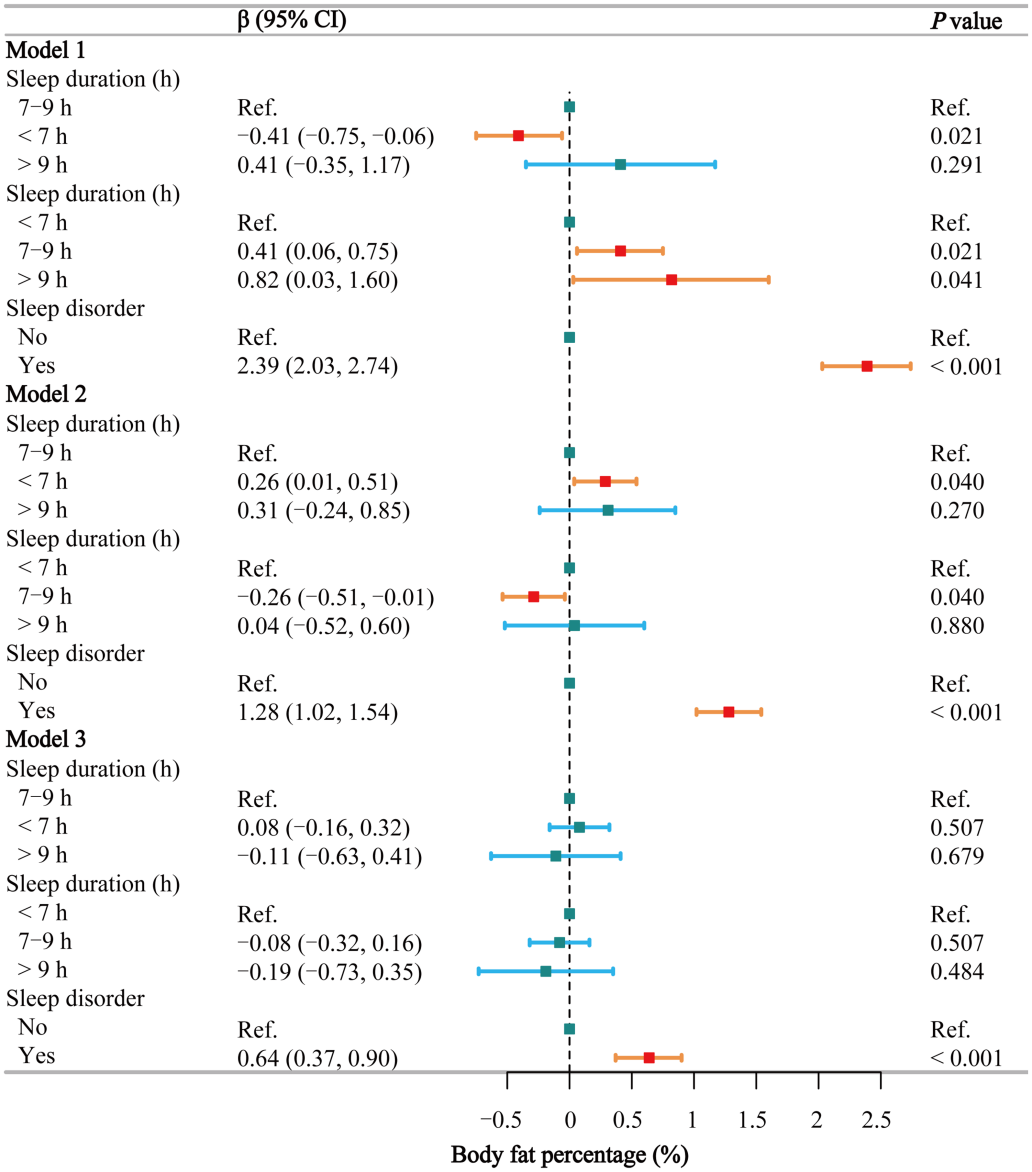
Associations between sleep behaviors and body fat percentage. Model 1 was unadjusted, Model 2 adjusted for gender, age, and race, and Model 3 adjusted for all covariates, including education level, marital status, poverty-income ratio, alcohol consumption, smoking, energy intake, hypertension, diabetes, stroke, depression, thyroid disease, and cancer. CI, confidence interval; h, hour; Ref, reference. *p* < 0.05 was considered statistically significant.

Figure 5 shows that in the fully adjusted model, sleep duration is not significantly associated with body fat percentage (*p* > 0.05), whereas sleep disorders are positively associated with body fat percentage (β = 0.64, 95% CI: 0.37–0.90). Smoothing curve fitting (Supplementary Figure S1D) and threshold effect analyses (Supplementary Table S14) indicate a segmented effect in males (*P* for log-likelihood = 0.045), with a positive correlation when sleep duration is less than 8.5 h and no significant association beyond this point. In females, sleep duration is not significantly associated with body fat percentage. Detailed subgroup analysis results on the effects of sleep duration (Supplementary Table S15) and sleep disorders (Supplementary Table S16) on body fat percentage are showing that sleep disorders have a more pronounced correlation. Additionally, no significant interaction (*p* = 0.268) was found between sleep duration and sleep disorders (Supplementary Table S17).

## Discussion

The relationship between sleep and body weight is complex and multifaceted, involving various physiological processes. Numerous studies have shown that both sleep quality and duration are critical for weight management and overall health. Our study demonstrated that individuals with short sleep duration (<7 h per night) exhibited significantly higher BMI, waist circumference, and lean body mass compared to those with normal sleep duration (7–9 h). These associations remained significant after adjusting for multiple confounders. Furthermore, gender-stratified analyses revealed that the negative relationship between sleep duration and BMI was more pronounced in males, whereas a segmented effect was observed in females, with sleep durations exceeding 12 h showing a slight positive correlation with BMI. Additionally, individuals with self-reported sleep disorders exhibited significantly higher BMI, waist circumference, lean body mass, and body fat percentage across all models, suggesting that both sleep duration and sleep quality play crucial roles in body composition. However, no significant interaction effects were observed between sleep duration and sleep disorders, indicating that these two factors may independently contribute to adiposity risk.

There is growing evidence that insufficient sleep is linked to negative health outcomes (7, 25, 26). Modern societal changes have led to reduced sleep duration and quality (27). Recent research has focused on the association between insufficient sleep and weight gain. Research has consistently found that insufficient sleep correlates with weight gain and a higher risk of obesity (28–33). For example, a meta-analysis by Cappuccio et al. (30), which included 36 studies encompassing 30,002 children and 604,509 adults, found that short sleep duration was associated with a higher risk of obesity. The risk of obesity was 1.89 times higher in children (*p* < 0.001) and 1.55 times higher in adults (*p* < 0.001) who slept less. Subsequent studies by Magee and Hale (31) and Ruan et al. (32) reported similar findings, noting an association between short sleep duration and weight gain. Consistently, our study also demonstrated positive correlations between short sleep duration and BMI (β = 0.56, 95% CI: 0.36–0.76), waist circumference (β = 0.90, 95% CI: 0.43–1.37), and lean body mass (β = 0.70, 95% CI: 0.32–1.07). These findings, in conjunction with the aforementioned studies, underscore the impact of short sleep duration on weight gain and the prevalence of obesity. While numerous studies mentioned above have reported that short sleep duration is associated with increased adiposity, some investigations have shown inconsistent findings or even a U-shaped relationship, particularly when accounting for subgroup differences such as gender, age, or socioeconomic status. For example, an analysis of the National Health Interview Survey data in the United States by Jean-Louis et al. (34) found that both insufficient sleep (<7 h) and excessive sleep (>8 h) were associated with an increased risk of obesity. Additionally, a study by Grandner et al. (35) highlighted that the relationship between sleep duration and BMI varies across different age groups, demonstrating a U-shaped pattern in middle-aged populations. Discrepancies in the literature may stem from differences in study designs, the use of self-reported sleep measures versus objective assessments, and varying adjustments for confounding factors. But the causal mechanisms underlying these relationships remain a topic of debate.

Good sleep is characterized not only by adequate duration but also by high quality. Research indicates that sufficient sleep duration and good sleep quality are more effective in promoting weight loss (36–45). Our results show that individuals with sleep disturbances have higher BMI (β = 0.93, 95% CI: 0.72–1.13), waist circumference (β = 2.40, 95% CI: 1.92–2.88), lean body mass (β = 0.71, 95% CI: 0.30–1.12), and body fat percentage (β = 0.64, 95% CI: 0.37–0.90). Although fewer studies have examined the impact of sleep disorders on weight and obesity compared to sleep duration, our results highlight the significant influence of sleep quality on these parameters. Similarly, Shade et al. conducted a weight loss intervention study to assess the impact of sleep quality on weight loss, finding that individuals who lost 5% or more of their body weight reported better sleep quality and fewer sleep disturbances (42).

The mechanisms through which sleep influences weight are not entirely understood, but research suggests they may involve imbalances in certain endocrine substances. A large cross-sectional study indicated that insufficient sleep is associated with higher levels of ghrelin and lower levels of leptin (46). Ghrelin and leptin play key roles in regulating appetite, food intake, and energy metabolism (47). Ghrelin not only increases ceramide expression, thus promoting appetite (48, 49), but also conserves fat and inhibits lipid oxidation simultaneously (50). *In vitro* experiments have demonstrated that ghrelin exerts anti-lipolytic effects in isolated adipocytes and muscle cells (51). Ghrelin is primarily found in the stomach’s fundus. Clinically, in some cases of non-morbid obesity, sleeve gastrectomy has resulted in an average BMI reduction of 5 kg/m^2^ within 2 years post-surgery (52). The level of leptin in the body is proportional to fat mass (53). Research has shown that the GH-IGF-1 axis, a major endocrine regulatory mechanism in anorexia nervosa, has IGF-1 levels that are proportional to leptin levels (54). This implies that when sleep duration is insufficient, a reduction in leptin levels can inhibit the regulation of the GH-IGF-1 axis, thereby increasing the desire for energy intake.

Moreover, insufficient sleep has been shown to disrupt the hypothalamic–pituitary–adrenal axis, leading to elevated cortisol levels—particularly at night or following sleep periods. Chronic sleep deprivation, attenuates the cortisol awakening response. Both mechanisms have been linked to downstream effects on inflammation and metabolic regulation, which are closely associated with fat storage (55–57). In addition to hormonal and metabolic factors, behavioral changes also play a critical role. Studies have shown that while shorter sleep duration may increase energy expenditure, it is often accompanied by higher energy intake (58–61), as prolonged wakefulness provides more opportunities for food consumption (62, 63). Our findings further support this evidence. Furthermore, individuals with shorter sleep duration tend to spend more time in sedentary activities, further exacerbating the risk of obesity (20).

Additionally, sleep disorder can affect the body’s circadian rhythms, leading to metabolic disturbances. Changes in sleep patterns can impact glucose-insulin metabolism, the activity of the hypothalamic–pituitary–adrenal axis, and levels of gut peptides (64). Individuals with disrupted circadian rhythms may exhibit higher appetites, particularly for protein-rich and sweet foods, compared to those with regular sleep patterns (65–67). A questionnaire survey on children’s sleep duration revealed that children with reduced sleep time were more likely to develop unhealthy eating habits, such as binge eating (68), and more likely to consume sugar and sugar-sweetened beverages, further contributing to weight gain and obesity (67). This suggests that the consequences of changing sleep patterns are multifaceted, including insulin resistance, nutritional metabolism disorders, imbalances in hunger and satiety, and potential weight gain and obesity (69).

Our findings underscore the importance of addressing sleep behaviors as part of obesity prevention and management strategies. Clinicians should consider screening for sleep disturbances in individuals with overweight or obesity, particularly those with short sleep duration or diagnosed sleep disorders. Public health initiatives should promote adequate sleep duration (7–9 h per night) as a key component of a healthy lifestyle, alongside diet and physical activity. Targeted interventions for vulnerable populations, such as women and individuals with limited access to healthcare, could help reduce obesity-related health disparities. Sleep should be recognized as a modifiable risk factor in clinical weight management strategies, clinicians should consider patients’ sleep behaviors in routine obesity treatment. Future research should focus on elucidating the biological mechanisms linking sleep to adiposity and evaluating the effectiveness of sleep-focused interventions in reducing obesity risk.

### Strengths and limitations

Unlike previous research that primarily focused on the association between sleep duration and general measures of adiposity such as BMI, our study expands the literature by examining a comprehensive set of adiposity indices—including waist circumference, lean body mass, and body fat percentage—using nationally representative NHANES data from 2011 to 2018. This dataset not only provides robust anthropometric measurements but also includes detailed assessments of sleep disorders, allowing us to simultaneously evaluate the independent effects of both sleep duration and sleep quality on body composition. Additionally, our use of gender-stratified smoothing curves and threshold analyses reveals nuanced, sex-specific associations that further our understanding of the complex interplay between sleep behaviors and adiposity. These methodological enhancements provide novel insights that can inform targeted public health interventions and clinical practices, thereby advancing the field beyond the scope of prior studies.

This study has several limitations. Firstly, the cross-sectional design prevents us from establishing causality between sleep behaviors and adiposity indices. Secondly, the data on sleep duration and sleep disorders were self-reported, which could introduce recall bias. Despite this, self-reported data in large population-based studies often provide reliable estimates. Third, although we adjusted for key socioeconomic indicators such as poverty-income ratio, education level, and marital status, we recognize that these may not fully capture the broader impact of socioeconomic disparities, healthcare access, and psychological stressors on both sleep behaviors and adiposity indices. Limited healthcare access may contribute to undiagnosed or untreated sleep disorders, while chronic stress related to financial instability or job insecurity could independently affect both sleep quality and metabolic outcomes. Additionally, while we adjusted for depression using PHQ-9, other mental health conditions—such as anxiety or chronic stress—may also play a role in shaping sleep patterns and obesity risk. Fourth, we acknowledge that some residual confounding may still exist due to unmeasured variables, such as genetic predispositions. By highlighting these factors, we aimed to include the most relevant confounders available in NHANES to minimize bias. And emphasize the need for future research that incorporates longitudinal data and more granular measures of healthcare access and psychosocial stressors.

## Conclusion

Cumulatively, the findings of this investigation underscore the association between brief sleep durations and sleep disorders with an elevated adiposity burden. Clinicians should consider integrating sleep assessments into routine obesity management. For patients with short sleep duration or sleep disorders, interventions such as cognitive behavioral therapy for insomnia, sleep hygiene education, or referral to sleep specialists could complement dietary and exercise recommendations. Concurrently, it is imperative for subsequent scholarly endeavors to delve deeper into the intricate causal dynamics or reciprocal associations that exist between sleep patterns and indices of adiposity, as well as to elucidate the foundational biological processes at play.

## Data Availability

The original contributions presented in the study are included in the article/Supplementary material, further inquiries can be directed to the corresponding author.

## Footnotes

^1^ https://www.cdc.gov/nchs/nhanes/index.htm

^2^ http://www.r-project.org

^3^ http://www.empowerstats.com
