# Supplementary material for: Association between sleep behaviors and adiposity indices among U.S. adults: a cross-sectional study
Source: Front Nutr. 2025 Mar 14;12:1526422. doi: 10.3389/fnut.2025.1526422 (PMC11949804; doi:10.3389/fnut.2025.1526422)
Supplement: Supplementary file 1 [file Table_1.DOCX]

**Supplementary Materials**

**Supplementary Figure 1.** The nonlinear association analyzed by the smooth curve fitting method between sleep duration and BMI (A), waist circumference (B), lean mass (C) and body fat percentage (D) of participants in the general U.S. population.

**Supplementary Table 1.** Characteristics of participants by categories of weekday sleep duration and sleep pattern status (weighted): NHANES 2011−2018

**Supplementary Table 2.** Threshold effect analysis of sleep duration on BMI.

**Supplementary Table 3.** Stratified logistic regression analysis was used to identify variables that affect the correlation between sleep duration and BMI.

**Supplementary Table 4.** Stratified logistic regression analysis was used to identify variables that affect the correlation between sleep disorder and BMI.

**Supplementary Table 5.** Examination of the interaction between sleep duration and sleep disorder on BMI.

**Supplementary Table 6.** Threshold effect analysis of sleep duration on WC.

**Supplementary Table 7.** Stratified logistic regression analysis was used to identify variables that affect the correlation between sleep duration and WC.

**Supplementary Table 8.** Stratified logistic regression analysis was used to identify variables that affect the correlation between sleep disorder and WC.

**Supplementary Table 9.** Examination of the interaction between sleep duration and sleep disorder on WC.

**Supplementary Table 10.** Threshold effect analysis of sleep duration on lean mass.

**Supplementary Table 11.** Stratified logistic regression analysis was used to identify variables that affect the correlation between sleep duration and lean mass.

**Supplementary Table 12.** Stratified logistic regression analysis was used to identify variables that affect the correlation between sleep disorder and lean mass.

**Supplementary Table 13.** Examination of the interaction between sleep duration and sleep disorder on lean mass.

**Supplementary Table 14.** Threshold effect analysis of sleep duration on body fat percentage.

**Supplementary Table 15.** Stratified logistic regression analysis was used to identify variables that affect the correlation between sleep duration and body fat percentage.

**Supplementary Table 16.** Stratified logistic regression analysis was used to identify variables that affect the correlation between sleep disorder and body fat percentage.

**Supplementary Table 17.** Examination of the interaction between sleep duration and sleep disorder on body fat percentage.


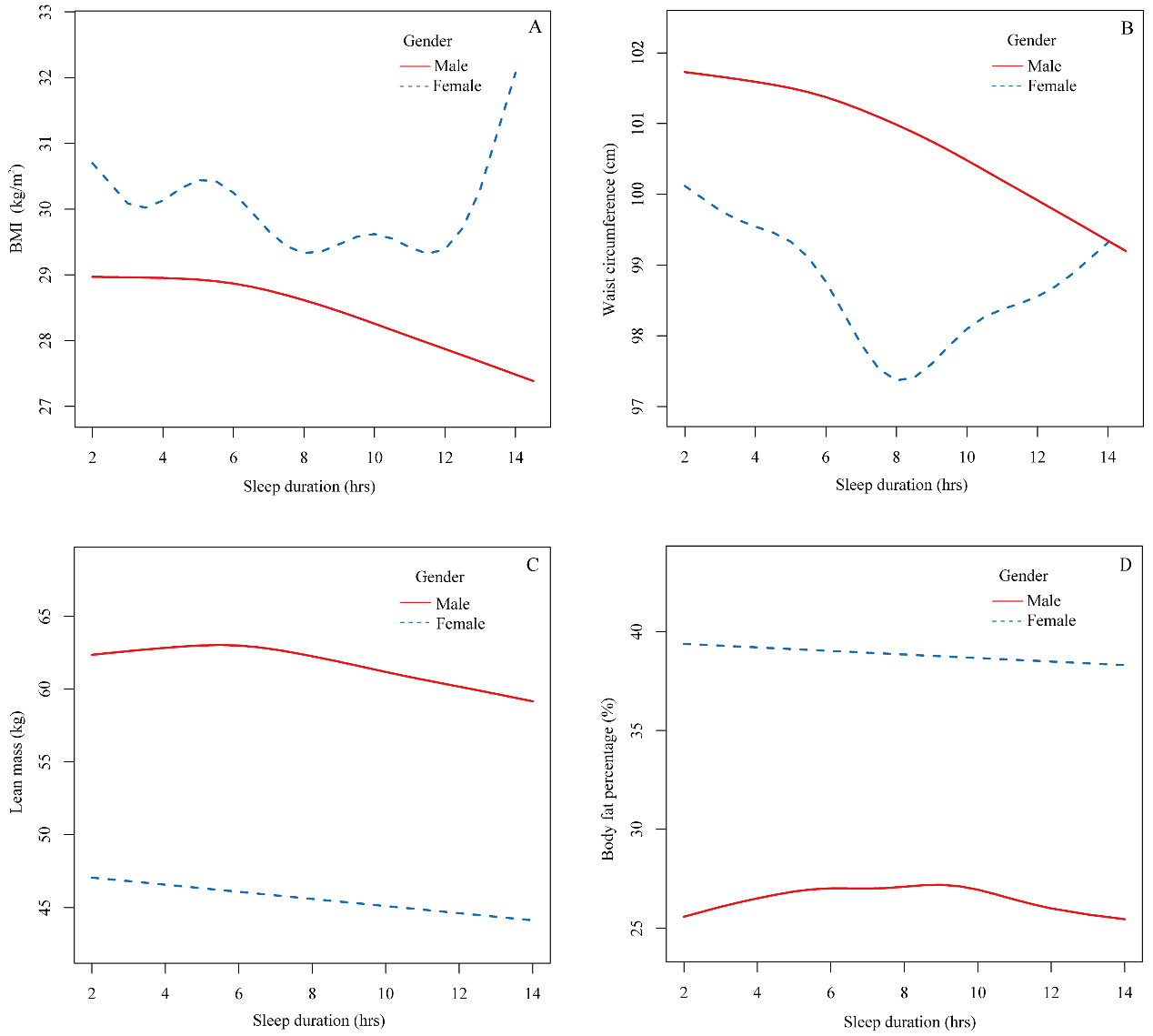


**Supplementary Figure 1.** The nonlinear association analyzed by the smooth curve fitting method between sleep duration and BMI (A), waist circumference (B), lean mass (C) and body fat percentage (D) of participants in the general U.S. population. The analysis was adjusted by age, gender, race, education level, marital status, poverty income ratio, physical activity, sedentariness, alcohol, smoking, energy intake, hypertension, diabetes, stroke, depression, thyroid disease, cancer or malignancy.

**Supplementary Table 1.** Characteristics of participants by categories of weekday sleep duration and sleep pattern status (weighted): NHANES 2011−2018

|  | **< 7 h** | | **7−9 h** | | **> 9 h** | | ***P* value** | | **Healthy** | | **Sleep disorder** | | ***P* value** | |
| --- | --- | --- | --- | --- | --- | --- | --- | --- | --- | --- | --- | --- | --- | --- |
| **Gender (%)** |  | |  | |  | | < 0.001 | |  | |  | | < 0.001 | |
| Male | 33502943.61 (53.23) | | 67348310.09 (47.84) | | 5256137.01 (41.19) | |  | | 78181287.03 (51.55) | | 27926103.69 (43.09) | |  | |
| Female | 29442472.30 (46.77) | | 73428496.77 (52.16) | | 7506108.16 (58.81) | |  | | 73490983.51 (48.45) | | 36886093.72 (56.91) | |  | |
| **Age (years, median [IQR])** | 46.00 [34.00, 58.00] | | 48.00 [33.00, 61.00] | | 53.00 [30.00, 68.00] | | < 0.001 | | 45.00 [31.00, 59.00] | | 52.00 [39.00, 63.00] | | < 0.001 | |
| **Race (%)** |  | |  | |  | | < 0.001 | |  | |  | | < 0.001 | |
| Mexican American | 5548583.73 (8.81) | | 11872746.14 (8.43) | | 1233698.52 (9.67) | |  | | 15199834.07 (10.02) | | 3455194.31 (5.33) | |  | |
| Other Hispanic | 4647702.81 (7.38) | | 8326087.90 (5.91) | | 795304.64 (6.23) | |  | | 10795656.77 (7.12) | | 2973438.57 (4.59) | |  | |
| Non-Hispanic White | 37104381.89 (58.95) | | 95760746.45 (68.02) | | 7869970.80 (61.67) | |  | | 93284093.92 (61.50) | | 47451005.23 (73.21) | |  | |
| Non-Hispanic Black | 10175308.62 (16.17) | | 12169568.68 (8.64) | | 1756001.00 (13.76) | |  | | 17796996.28 (11.73) | | 6303882.02 (9.73) | |  | |
| Non-Hispanic Asian | 3205517.41 (5.09) | | 8013347.73 (5.69) | | 636430.83 (4.99) | |  | | 10097122.47 (6.66) | | 1758173.50 (2.71) | |  | |
| Other Race | 2263921.45 (3.60) | | 4634309.95 (3.29) | | 470839.39 (3.69) | |  | | 4498567.01 (2.97) | | 2870503.77 (4.43) | |  | |
| **Education level (%)** |  | |  | |  | | < 0.001 | |  | |  | | < 0.001 | |
| Less than 9th grade | 2966158.00 (4.71) | | 6337193.85 (4.50) | | 1003243.48 (7.86) | |  | | 7986492.62 (5.27) | | 2320102.70 (3.58) | |  | |
| 9-11th grade | 6614156.46 (10.51) | | 11282710.36 (8.01) | | 2101921.78 (16.47) | |  | | 14540989.41 (9.59) | | 5457799.19 (8.42) | |  | |
| High school graduate | 15617486.93 (24.81) | | 29378835.66 (20.87) | | 3615460.66 (28.33) | |  | | 33618386.09 (22.17) | | 14993397.15 (23.13) | |  | |
| Some college or AA degree | 22125760.79 (35.15) | | 43723240.66 (31.06) | | 3732790.26 (29.25) | |  | | 47157445.91 (31.09) | | 22424345.80 (34.60) | |  | |
| College graduate or above | 15616986.38 (24.81) | | 50015998.65 (35.53) | | 2296777.02 (18.00) | |  | | 48319775.12 (31.86) | | 19609986.94 (30.26) | |  | |
| Missing | 4867.35 (0.01) | | 38827.67 (0.03) | | 12051.97 (0.09) | |  | | 49181.38 (0.03) | | 6565.61 (0.01) | |  | |
| **Marital status (%)** |  | |  | |  | | < 0.001 | |  | |  | | < 0.001 | |
| Married | 32431422.76 (51.52) | | 80398522.14 (57.11) | | 5036854.01 (39.47) | |  | | 84186611.96 (55.51) | | 33680186.95 (51.97) | |  | |
| Widowed | 2952796.55 (4.69) | | 7401167.17 (5.26) | | 1200520.35 (9.41) | |  | | 6829815.38 (4.50) | | 4724668.69 (7.29) | |  | |
| Divorced | 7624248.69 (12.11) | | 13174458.64 (9.36) | | 1498463.08 (11.74) | |  | | 13333953.44 (8.79) | | 8963216.96 (13.83) | |  | |
| Separated | 2114713.96 (3.36) | | 2778839.78 (1.97) | | 411675.62 (3.23) | |  | | 3410597.89 (2.25) | | 1894631.47 (2.92) | |  | |
| Never married | 12252899.85 (19.47) | | 25629062.16 (18.21) | | 3007301.72 (23.56) | |  | | 30579535.91 (20.16) | | 10309727.81 (15.91) | |  | |
| Living with partner | 5557640.81 (8.83) | | 11382325.98 (8.09) | | 1602322.54 (12.56) | |  | | 13308130.97 (8.77) | | 5234158.35 (8.08) | |  | |
| Missing | 11693.31 (0.02) | | 12431.00 (0.01) | | 5107.85 (0.04) | |  | | 23624.99 (0.02) | | 5607.17 (0.01) | |  | |
| **Poverty income ratio (%)** |  | |  | |  | | < 0.001 | |  | |  | | 0.102 | |
| ≤ 1.3 | 14659712.10 (23.29) | | 25424100.27 (18.06) | | 4401194.42 (34.49) | |  | | 30877952.97 (20.36) | | 13607053.81 (20.99) | |  | |
| > 1.3 and ≤ 3.5 | 21435505.28 (34.05) | | 44772897.17 (31.80) | | 4446286.37 (34.84) | |  | | 49427464.14 (32.59) | | 21227224.68 (32.75) | |  | |
| > 3.5 | 22086331.78 (35.09) | | 60070092.98 (42.67) | | 2793389.85 (21.89) | |  | | 59202460.74 (39.03) | | 25747353.88 (39.73) | |  | |
| Missing | 4763866.75 (7.57) | | 10509716.44 (7.47) | | 1121374.53 (8.79) | |  | | 12164392.69 (8.02) | | 4230565.03 (6.53) | |  | |
| **Physical activity (MET-h, median [IQR])** | 13.30 [5.10, 30.00] | | 10.70 [4.00, 24.00] | | 11.73 [4.00, 24.00] | | < 0.001 | | 12.00 [4.30, 27.00] | | 10.00 [4.00, 23.00] | | < 0.001 | |
| **Sedentary time (hours, median [IQR])** | 6.00 [4.00, 8.00] | | 6.00 [4.00, 8.00] | | 6.00 [4.00, 8.00] | | 0.013 | | 6.00 [4.00, 8.00] | | 6.00 [4.00, 9.00] | | < 0.001 | |
| **Alcohol (%)** |  | |  | |  | | < 0.001 | |  | |  | | 0.833 | |
| No | 20551919.34 (32.65) | | 51357282.98 (36.48) | | 3860313.29 (30.25) | |  | | 52979101.84 (34.93) | | 22790413.77 (35.16) | |  | |
| Yes | 23158881.02 (36.79) | | 49917984.01 (35.46) | | 4056938.54 (31.79) | |  | | 53864390.11 (35.51) | | 23269413.46 (35.90) | |  | |
| Missing | 19234615.55 (30.56) | | 39501539.87 (28.06) | | 4844993.34 (37.96) | |  | | 44828778.58 (29.56) | | 18752370.17 (28.93) | |  | |
| **Smoking (%)** |  | |  | |  | | < 0.001 | |  | |  | | < 0.001 | |
| Non-users | 32696799.18 (51.94) | | 82630176.26 (58.70) | | 6676268.93 (52.31) | |  | | 92113110.17 (60.73) | | 29890134.19 (46.12) | |  | |
| Current smoking | 15865607.18 (25.21) | | 22170881.42 (15.75) | | 3028632.59 (23.73) | |  | | 26076287.23 (17.19) | | 14988833.95 (23.13) | |  | |
| Past smoking | 14337201.79 (22.78) | | 35935623.43 (25.53) | | 3049237.07 (23.89) | |  | | 33423770.62 (22.04) | | 19898291.68 (30.70) | |  | |
| Missing | 45807.77 (0.07) | | 40125.75 (0.03) | | 8106.58 (0.06) | |  | | 59102.52 (0.04) | | 34937.57 (0.05) | |  | |
| **Energy intake (kcal/d, median [IQR])** | 2012.48 [1531.00, 2625.59] | 1984.71 [1545.50, 2541.50] | | 1797.43 [1376.02, 2353.99] | | < 0.001 | | 2006.50 [1557.50, 2580.00] | | 1926.95 [1478.33, 2497.00] | | < 0.001 | |  |
| **Weight (kg, median [IQR])** | 82.10 [69.10, 98.20] | 79.20 [67.12, 93.90] | | 78.20 [65.60, 92.80] | | < 0.001 | | 78.90 [67.00, 93.20] | | 83.07 [69.30, 99.30] | | < 0.001 | |  |
| **BMI (kg/m^2^, median [IQR])** | 28.50 [24.80, 33.50] | 27.80 [24.10, 32.50] | | 28.18 [24.40, 32.78] | | < 0.001 | | 27.70 [24.10, 32.00] | | 29.20 [25.00, 34.50] | | < 0.001 | |  |
| **Waist (cm, median [IQR])** | 99.20 [88.60, 110.90] | 97.90 [87.20, 109.20] | | 98.55 [88.00, 111.10] | | < 0.001 | | 97.00 [86.60, 107.90] | | 102.00 [90.70, 114.10] | | < 0.001 | |  |
| **Lean mass (kg, median [IQR])** | 56.20 [45.90, 65.40] | 53.20 [44.00, 63.10] | | 51.50 [43.80, 61.07] | | < 0.001 | | 53.90 [44.40, 63.50] | | 54.30 [44.80, 65.00] | | 0.044 | |  |
| **Body fat percentage (%, median [IQR])** | 32.10 [26.10, 39.30] | 32.60 [26.90, 39.60] | | 33.80 [26.50, 40.90] | | 0.196 | | 31.70 [26.00, 38.90] | | 34.50 [28.50, 41.30] | | < 0.001 | |  |
| **Hypertension (%)** |  | |  | |  | | < 0.001 | |  | |  | | < 0.001 | |
| No | 37345051.93 (59.33) | | 89201173.28 (63.36) | | 6782300.71 (53.14) | |  | | 101380190.20 (66.84) | | 31948335.72 (49.29) | |  | |
| Yes | 25550024.91 (40.59) | | 51467604.65 (36.56) | | 5967108.94 (46.76) | |  | | 50152291.86 (33.07) | | 32832446.64 (50.66) | |  | |
| Missing | 50339.07 (0.08) | | 108028.94 (0.08) | | 12835.52 (0.10) | |  | | 139788.48 (0.09) | | 31415.05 (0.05) | |  | |
| **Diabetes (%)** |  | |  | |  | | < 0.001 | |  | |  | | < 0.001 | |
| No | 53233723.95 (84.57) | | 122480563.73 (87.00) | | 10139657.01 (79.45) | |  | | 133970609.53 (88.33) | | 51883335.16 (80.05) | |  | |
| Yes | 8262212.63 (13.13) | | 16152489.01 (11.47) | | 2421473.80 (18.97) | |  | | 15421859.75 (10.17) | | 11414315.68 (17.61) | |  | |
| Borderline | 1449479.34 (2.30) | | 2143754.12 (1.52) | | 201114.37 (1.58) | |  | | 2279801.25 (1.50) | | 1514546.57 (2.34) | |  | |
| **Depression (%)** |  | |  | |  | | < 0.001 | |  | |  | | < 0.001 | |
| No | 51079487.32 (81.15) | | 123264629.69 (87.56) | | 9891994.55 (77.51) | |  | | 134308521.12 (88.55) | | 49927590.44 (77.03) | |  | |
| Yes | 7640988.36 (12.14) | | 9006019.69 (6.40) | | 1917000.29 (15.02) | |  | | 6608307.02 (4.36) | | 11955701.32 (18.45) | |  | |
| Missing | 4224940.24 (6.71) | | 8506157.47 (6.04) | | 953250.33 (7.47) | |  | | 10755442.40 (7.09) | | 2928905.64 (4.52) | |  | |
| **Stroke (%)** |  | |  | |  | | < 0.001 | |  | |  | | < 0.001 | |
| No | 61258104.07 (97.32) | | 137352224.68 (97.57) | | 12068024.49 (94.56) | |  | | 148660854.50 (98.01) | | 62017498.75 (95.69) | |  | |
| Yes | 1618784.41 (2.57) | | 3350197.27 (2.38) | | 690830.38 (5.41) | |  | | 2954973.29 (1.95) | | 2704838.76 (4.17) | |  | |
| Missing | 68527.43 (0.11) | | 74384.92 (0.05) | | 3390.29 (0.03) | |  | | 56442.75 (0.04) | | 89859.89 (0.14) | |  | |
| **Thyroid disease (%)** |  | |  | |  | | 0.074 | |  | |  | | < 0.001 | |
| No | 56087196.51 (89.10) | | 124537732.79 (88.46) | | 11078078.72 (86.80) | |  | | 138201423.90 (91.12) | | 53501584.14 (82.55) | |  | |
| Yes | 6678306.84 (10.61) | | 16029836.41 (11.39) | | 1672631.25 (13.11) | |  | | 13337980.39 (8.79) | | 11042794.11 (17.04) | |  | |
| Missing | 179912.56 (0.29) | | 209237.66 (0.15) | | 11535.20 (0.09) | |  | | 132866.25 (0.09) | | 267819.16 (0.41) | |  | |
| **Cancer or malignancy (%)** |  | |  | |  | | 0.002 | |  | |  | | < 0.001 | |
| No | 57401961.93 (91.19) | | 125246414.34 (88.97) | | 11080402.72 (86.82) | |  | | 138546475.41 (91.35) | | 55182303.57 (85.14) | |  | |
| Yes | 5468901.56 (8.69) | | 15483391.34 (11.00) | | 1681842.45 (13.18) | |  | | 13037491.31 (8.60) | | 9596644.04 (14.81) | |  | |
| Missing | 74552.42 (0.12) | | 47001.18 (0.03) | | 0.00 (0.00) | |  | | 88303.82 (0.06) | | 33249.79 (0.05) | |  | |

IQR, interquartile range; kg, kilogram; cm, centimeter; n, number; BMI, body mass index.

**Supplementary Table 1.** Threshold effect analysis of sleep duration on BMI.

|  | **Male** |  | **Female** |  |
| --- | --- | --- | --- | --- |
|  | **β (95% CI) *P* value** | **β (95% CI) *P* value** | | |
| **One linear model** | −0.13 (−0.21, −0.05) 0.002 | −0.20 (−0.30, −0.11)  < 0.001 | | |
| **Piecewise model** |  |  | | |
| Inflection point (h) | 5, 12 | 5, 12 | | |
| Model 1: sleep duration < 5 | −0.28 (−0.75, 0.20) 0.253 | 0.50 (−0.09, 1.08)  0.096 | | |
| Model 2: 5 ≤ sleep duration ≤ 12 | −0.12 (−0.21, −0.03) 0.012 | −0.29 (−0.40, −0.18)  < 0.001 | | |
| Model 3: sleep duration > 12 | −4.48 (−10.62, 1.65) 0.152 | 10.77 (5.27, 16.28)  < 0.001 | | |
| Model 1 and 2 effect differences | −0.16 (−0.66, 0.35) 0.540 | 0.79 (0.16, 1.41)  0.014 | | |
| Model 3 and 2 effect differences | 4.37 (−10.50, 1.77) 0.163 | 11.06 (5.56, 16.57)  < 0.001 | | |
| *P* for logarithmic likelihood ratio test | 0.106 | < 0.001 | | |

BMI, body mass index; h, hour; CI, confidence interval.

**Supplementary Table 2.** Stratified logistic regression analysis was used to identify variables that affect the correlation between sleep duration and BMI.

| **Subgroup** | ***n*** | **7**−**9 h** | **< 7 h** | |  | **> 9 h** | |  |
| --- | --- | --- | --- | --- | --- | --- | --- | --- |
|  |  |  | **β (95% CI)** | ***P* value** | | **β (95% CI)** | ***P* value** | |
| **Age** |  |  |  |  | |  |  | |
| 20−34 | 4681 | Ref | 0.33 (−0.12, 0.78) | 0.154 | | −0.75 (−1.55, 0.04) | 0.063 | |
| 35−49 | 5029 | Ref | 0.53 (0.14, 0.92) | 0.007 | | 0.73 (−0.26, 1.73) | 0.147 | |
| 50−63 | 4954 | Ref | 0.29 (−0.08, 0.67) | 0.128 | | −0.07 (−0.93, 0.78) | 0.870 | |
| ≥ 64 | 5287 | Ref | 0.59 (0.21, 0.97) | 0.002 | | −0.06 (−0.59, 0.48) | 0.836 | |
| **Gender** |  |  |  |  | |  |  | |
| Male | 9825 | Ref | 0.16 (−0.08, 0.41) | 0.193 | | −0.49 (−1.02, 0.03) | 0.067 | |
| Female | 10126 | Ref | 0.76 (0.44, 1.08) | < 0.001 | | 0.04 (−0.52, 0.59) | 0.893 | |
| **Race** |  |  |  |  | |  |  | |
| Mexican American | 2708 | Ref | 0.62 (0.09, 1.15) | 0.021 | | 0.14 (−0.84, 1.11) | 0.784 | |
| Other Hispanic | 2098 | Ref | 0.37 (−0.19, 0.92) | 0.196 | | −0.06 (−1.20, 1.07) | 0.912 | |
| Non-Hispanic White | 7355 | Ref | 0.47 (0.13, 0.80) | 0.007 | | −0.00 (−0.65, 0.64) | 0.998 | |
| Non-Hispanic Black | 4507 | Ref | 0.55 (0.11, 0.99) | 0.015 | | −0.68 (−1.54, 0.17) | 0.116 | |
| Non-Hispanic Asian | 2574 | Ref | 0.30 (−0.09, 0.68) | 0.129 | | −0.23 (−0.98, 0.53) | 0.557 | |
| Other Race | 709 | Ref | −0.23 (−1.33, 0.88) | 0.686 | | −1.58 (−3.68, 0.52) | 0.141 | |
| **Education** |  |  |  |  | |  |  | |
| Less than 9th grade | 1812 | Ref | 0.39 (−0.19, 0.98) | 0.186 | | −0.28 (−1.20, 0.64) | 0.547 | |
| 9-11th grade | 2514 | Ref | 0.20 (−0.35, 0.76) | 0.469 | | −0.26 (−1.10, 0.58) | 0.547 | |
| High school graduate | 4435 | Ref | 0.38 (−0.06, 0.82) | 0.093 | | −0.22 (−1.00, 0.57) | 0.589 | |
| Some college or AA degree | 6151 | Ref | 0.40 (0.02, 0.77) | 0.038 | | −0.16 (−0.92, 0.61) | 0.684 | |
| College graduate or above | 5026 | Ref | 0.60 (0.22, 0.99) | 0.002 | | 0.20 (−0.69, 1.09) | 0.660 | |
| Missing | 13 | Ref | - | - | |  |  | |
| **Marital** |  |  |  |  | |  |  | |
| Married | 10107 | Ref | 0.53 (0.26, 0.80) | < 0.001 | | −0.02 (−0.61, 0.58) | 0.956 | |
| Widowed | 1403 | Ref | 0.95 (0.18, 1.72) | 0.015 | | 0.05 (−1.04, 1.14) | 0.928 | |
| Divorced | 2181 | Ref | −0.22 (−0.79, 0.35) | 0.446 | | −0.52 (−1.61, 0.57) | 0.348 | |
| Separated | 689 | Ref | 0.52 (−0.67, 1.71) | 0.392 | | −1.30 (−3.41, 0.81) | 0.228 | |
| Never married | 3858 | Ref | 0.54 (0.04, 1.04) | 0.034 | | −0.34 (−1.21, 0.53) | 0.446 | |
| Living with partner | 1704 | Ref | 0.23 (−0.51, 0.97) | 0.545 | | 0.55 (−0.64, 1.74) | 0.362 | |
| Missing | 9 | Ref | - | - | |  |  | |
| **Poverty income ratio** |  |  |  |  | |  |  | |
| ≤ 1.3 | 5867 | Ref | 0.60 (0.21, 0.98) | 0.002 | | 0.16 (−0.44, 0.77) | 0.597 | |
| > 1.3 and ≤ 3.5 | 6745 | Ref | 0.32 (−0.03, 0.67) | 0.075 | | −0.13 (−0.79, 0.54) | 0.712 | |
| > 3.5 | 5471 | Ref | 0.49 (0.13, 0.85) | 0.007 | | −0.21 (−1.08, 0.66) | 0.642 | |
| Missing | 1868 | Ref | 0.16 (−0.51, 0.84) | 0.639 | | −0.54 (−1.74, 0.66) | 0.376 | |
| **Alcohol** |  |  |  |  | |  |  | |
| No | 6361 | Ref | 0.68 (0.34, 1.03) | < 0.001 | | 0.35 (−0.35, 1.04) | 0.327 | |
| Yes | 6282 | Ref | 0.21 (−0.16, 0.58) | 0.272 | | −0.50 (−1.26, 0.26) | 0.195 | |
| Missing | 7308 | Ref | 0.40 (0.07, 0.74) | 0.016 | | −0.13 (−0.70, 0.45) | 0.667 | |
| **Smoking** |  |  |  |  | |  |  | |
| Non-users | 11414 | Ref | 0.59 (0.32, 0.87) | <0.001 | | −0.39 (-0.91, 0.14) | 0.149 | |
| Current smoking | 3912 | Ref | 0.29 (−0.14, 0.72) | 0.182 | | 0.17 (−0.63, 0.98) | 0.674 | |
| Past smoking | 4610 | Ref | 0.34 (−0.07, 0.76) | 0.107 | | 0.28 (−0.51, 1.08) | 0.484 | |
| Missing | 15 | Ref | - | - | | - | - | |
| **Hypertension** |  |  |  |  | |  |  | |
| No | 11359 | Ref | 0.42 (0.16, 0.68) | 0.001 | | −0.16 (−0.69, 0.36) | 0.537 | |
| Yes | 8575 | Ref | 0.38 (0.06, 0.69) | 0.019 | | −0.10 (−0.66, 0.46) | 0.720 | |
| Missing | 17 | Ref | - | - | | - | - | |
| **Diabetes** |  |  |  |  | |  |  | |
| No | 16234 | Ref | 0.40 (0.18, 0.62) | < 0.001 | | −0.29 (−0.72, 0.15) | 0.193 | |
| Yes | 3348 | Ref | 0.85 (0.34, 1.35) | 0.001 | | 0.30 (−0.51, 1.11) | 0.468 | |
| Borderline | 369 | Ref | 0.85 (-0.64, 2.34) | 0.264 | | 2.48 (−0.77, 5.73) | 0.136 | |
| **Depression** |  |  |  |  | |  |  | |
| No | 16521 | Ref | 0.43 (0.21, 0.65) | < 0.001 | | −0.11 (−0.55, 0.32) | 0.611 | |
| Yes | 1849 | Ref | 0.75 (0.05, 1.45) | 0.036 | | −0.21 (−1.34, 0.92) | 0.712 | |
| Missing | 1581 | Ref | 0.74 (0.06, 1.42) | 0.034 | | 0.22 (−1.04, 1.48) | 0.734 | |
| **Stroke** |  |  |  |  | |  |  | |
| No | 19219 | Ref | 0.48 (0.28, 0.69) | < 0.001 | | −0.15 (−0.54, 0.25) | 0.468 | |
| Yes | 715 | Ref | −0.81 (−1.93, 0.31) | 0.156 | | −0.49 (−2.02, 1.03) | 0.525 | |
| Missing | 17 | Ref | - | - | | - | - | |
| **Thyroid disease** |  |  |  |  | |  |  | |
| No | 17835 | Ref | 0.50 (0.29, 0.71) | < 0.001 | | −0.04 (−0.45, 0.37) | 0.847 | |
| Yes | 2074 | Ref | −0.03 (−0.74, 0.68) | 0.927 | | −0.89 (−2.13, 0.35) | 0.161 | |
| Missing | 42 | Ref | - | - | | - | - | |
| **Cancer or malignancy** |  |  |  |  | |  |  | |
| No | 18086 | Ref | 0.52 (0.31, 0.73) | < 0.001 | | −0.15 (−0.56, 0.26) | 0.466 | |
| Yes | 1854 | Ref | −0.24 (−0.92, 0.45) | 0.502 | | −0.19 (−1.28, 0.91) | 0.738 | |
| Missing | 11 | Ref | - | - | | - | - | |
| **Physical activity** |  |  |  |  | |  |  | |
| 0.7−4 | 4374 | Ref | 0.04 (−0.41, 0.49) | 0.865 | | −0.70 (−1.54, 0.14) | 0.103 | |
| 4.1−10.7 | 3164 | Ref | 0.93 (0.43, 1.44) | < 0.001 | | 0.88 (−0.15, 1.91) | 0.094 | |
| 10.8−24 | 3774 | Ref | 0.62 (0.19, 1.06) | 0.005 | | 0.03 (−0.83, 0.89) | 0.945 | |
| 24.1−172 | 3673 | Ref | −0.23 (−0.67, 0.21) | 0.301 | | −1.65 (−2.62, −0.67) | < 0.001 | |
| Missing | 4966 | Ref | - | - | | - | - | |
| **Sedentary time** |  |  |  |  | |  |  | |
| 0−4 | 7394 | Ref | 0.20 (−0.11, 0.51) | 0.207 | | −0.59 (−1.18, −0.01) | 0.045 | |
| 4.1−6 | 4389 | Ref | 0.61 (0.18, 1.04) | 0.006 | | −0.27 (−1.05, 0.52) | 0.506 | |
| 6.1−8 | 3829 | Ref | 0.77 (0.32, 1.22) | < 0.001 | | 0.43 (−0.40, 1.26) | 0.309 | |
| 8.1−22 | 4223 | Ref | 0.42 (−0.05, 0.90) | 0.080 | | 0.50 (−0.56, 1.57) | 0.356 | |
| Missing | 116 | Ref | - | - | | - | - | |
| **Energy intake (kcal/d)** |  |  |  |  | |  |  | |
| 14−1462.5 | 4633 | Ref | 0.37 (−0.05, 0.79) | 0.087 | | −0.52 (−1.24, 0.20) | 0.154 | |
| 1462.6−1912 | 4645 | Ref | 0.28 (−0.15, 0.71) | 0.196 | | −0.52 (−1.30, 0.25) | 0.186 | |
| 1912.1−2482.1 | 4639 | Ref | 0.60 (0.19, 1.01) | 0.004 | | 0.95 (0.13, 1.78) | 0.023 | |
| 2482.2−10025 | 4639 | Ref | 0.57 (0.16, 0.98) | 0.006 | | −0.59 (−1.50, 0.33) | 0.208 | |
| Missing | 1395 | Ref | - | - | | - | - | |

The analysis was adjusted by age, gender, race, education level, marital status, poverty income ratio, physical activity, sedentariness, alcohol, smoking, energy intake, hypertension, diabetes, stroke, depression, thyroid disease, cancer or malignancy. BMI, body mass index; h, hour; n, number; CI, confidence interval; Ref, reference; d, day; MET, metabolic equivalent.

**Supplementary Table 3.** Stratified logistic regression analysis was used to identify variables that affect the correlation between sleep disorder and BMI.

| **Subgroup** | ***n*** | **Healthy** | **Sleep disorder** | |
| --- | --- | --- | --- | --- |
|  |  |  | **β (95% CI)** | ***P* value** |
| **Age** |  |  |  |  |
| 20−34 | 4681 | Ref | 0.44 (−0.08, 0.96) | 0.095 |
| 35−49 | 5029 | Ref | 1.19 (0.76, 1.61) | < 0.001 |
| 50−63 | 4954 | Ref | 0.93 (0.55, 1.31) | < 0.001 |
| ≥ 64 | 5287 | Ref | 0.54 (0.21, 0.86) | 0.001 |
| **Gender** |  |  |  |  |
| Male | 9825 | Ref | 1.13 (0.86, 1.40) | < 0.001 |
| Female | 10126 | Ref | 0.60 (0.30, 0.91) | < 0.001 |
| **Race** |  |  |  |  |
| Mexican American | 2708 | Ref | 1.23 (0.59, 1.88) | < 0.001 |
| Other Hispanic | 2098 | Ref | 1.06 (0.39, 1.73) | 0.002 |
| Non-Hispanic White | 7355 | Ref | 0.73 (0.41, 1.05) | < 0.001 |
| Non-Hispanic Black | 4507 | Ref | 1.04 (0.53, 1.56) | < 0.001 |
| Non-Hispanic Asian | 2574 | Ref | 0.45 (−0.03, 0.94) | 0.065 |
| Other Race | 709 | Ref | 0.84 (−0.25, 1.93) | 0.132 |
| **Education** |  |  |  |  |
| Less than 9th grade | 1812 | Ref | 1.29 (0.61, 1.96) | < 0.001 |
| 9-11th grade | 2514 | Ref | 1.52 (0.91, 2.13) | < 0.001 |
| High school graduate | 4435 | Ref | 1.00 (0.54, 1.45) | < 0.001 |
| Some college or AA degree | 6151 | Ref | 0.57 (0.18, 0.95) | 0.004 |
| College graduate or above | 5026 | Ref | 0.73 (0.37, 1.10) | < 0.001 |
| Missing | 13 | Ref | - | - |
| **Marital** |  |  |  |  |
| Married | 10107 | Ref | 0.63 (0.36, 0.91) | < 0.001 |
| Widowed | 1403 | Ref | 0.34 (−0.35, 1.02) | 0.338 |
| Divorced | 2181 | Ref | 1.27 (0.71, 1.83) | < 0.001 |
| Separated | 689 | Ref | 0.57 (−0.69, 1.84) | 0.374 |
| Never married | 3858 | Ref | 1.80 (1.26, 2.35) | < 0.001 |
| Living with partner | 1704 | Ref | 0.09 (−0.70, 0.88) | 0.827 |
| Missing | 9 | Ref | - | - |
| **Poverty income ratio** |  |  |  |  |
| ≤ 1.3 | 5867 | Ref | 0.94 (0.52, 1.36) | < 0.001 |
| > 1.3 and ≤ 3.5 | 6745 | Ref | 0.76 (0.39, 1.13) | < 0.001 |
| > 3.5 | 5471 | Ref | 0.72 (0.37, 1.07) | < 0.001 |
| Missing | 1868 | Ref | - | - |
| **Alcohol** |  |  |  |  |
| No | 6361 | Ref | 1.08 (0.74, 1.43) | < 0.001 |
| Yes | 6282 | Ref | 0.49 (0.10, 0.87) | 0.013 |
| Missing | 7308 | Ref | - | - |
| **Smoking** |  |  |  |  |
| Non-users | 11414 | Ref | 0.86 (0.57, 1.14) | < 0.001 |
| Current smoking | 3912 | Ref | 0.61 (0.16, 1.06) | 0.008 |
| Past smoking | 4610 | Ref | 0.97 (0.59, 1.36) | < 0.001 |
| Missing | 15 | Ref | - | - |
| **Hypertension** |  |  |  |  |
| No | 11359 | Ref | 0.29 (0.01, 0.56) | 0.042 |
| Yes | 8575 | Ref | 1.39 (1.09, 1.69) | < 0.001 |
| Missing | 17 | Ref | - | - |
| **Diabetes** |  |  |  |  |
| No | 16234 | Ref | 0.67 (0.44, 0.89) | < 0.001 |
| Yes | 3348 | Ref | 1.74 (1.26, 2.22) | < 0.001 |
| Borderline | 369 | Ref | 0.99 (−0.44, 2.43) | 0.176 |
| **Depression** |  |  |  |  |
| No | 16521 | Ref | 0.78 (0.56, 1.01) | < 0.001 |
| Yes | 1849 | Ref | 1.21 (0.48, 1.93) | 0.001 |
| Missing | 1581 | Ref | - | - |
| **Stroke** |  |  |  |  |
| No | 19219 | Ref | 0.82 (0.61, 1.03) | < 0.001 |
| Yes | 715 | Ref | 0.96 (−0.05, 1.97) | 0.062 |
| Missing | 17 | Ref | - | - |
| **Thyroid disease** |  |  |  |  |
| No | 17835 | Ref | 0.90 (0.69, 1.12) | < 0.001 |
| Yes | 2074 | Ref | 0.45 (−0.18, 1.09) | 0.163 |
| Missing | 42 | Ref | - | - |
| **Cancer or malignancy** |  |  |  |  |
| No | 18086 | Ref | 0.87 (0.65, 1.09) | <0.001 |
| Yes | 1854 | Ref | 0.65 (0.07, 1.24) | 0.029 |
| Missing | 11 | Ref | - | - |
| **Physical activity** |  |  |  |  |
| 0.7−4 | 4374 | Ref | 0.74 (0.31, 1.17) | < 0.001 |
| 4.1−10.7 | 3164 | Ref | 0.85 (0.35, 1.34) | < 0.001 |
| 10.8−24 | 3774 | Ref | 0.92 (0.47, 1.37) | < 0.001 |
| 24.1−172 | 3673 | Ref | 0.68 (0.19, 1.17) | 0.007 |
| Missing | 4966 | Ref | - | - |
| **Sedentary time** |  |  |  |  |
| 0−4 | 7394 | Ref | 0.50 (0.16, 0.84) | 0.004 |
| 4.1−6 | 4389 | Ref | 0.48 (0.05, 0.91) | 0.031 |
| 6.1−8 | 3829 | Ref | 0.94 (0.49, 1.40) | < 0.001 |
| 8.1−22 | 4223 | Ref | 1.49 (1.03, 1.95) | < 0.001 |
| Missing | 116 | Ref | - | - |
| **Energy intake (kcal/d)** |  |  |  |  |
| 14−1462.5 | 4633 | Ref | 0.90 (0.48, 1.32) | < 0.001 |
| 1462.6−1912 | 4645 | Ref | 0.70 (0.27, 1.13) | 0.001 |
| 1912.1−2482.1 | 4639 | Ref | 0.96 (0.55, 1.37) | < 0.001 |
| 2482.2−10025 | 4639 | Ref | 0.54 (0.10, 0.97) | 0.015 |
| Missing | 1395 | Ref | - | - |

The analysis was adjusted by age, gender, race, education level, marital status, poverty income ratio, physical activity, sedentariness, alcohol, smoking, energy intake, hypertension, diabetes, stroke, depression, thyroid disease, cancer or malignancy. BMI, body mass index; n, number; CI, confidence interval; Ref, reference; d, day; MET, metabolic equivalent.

**Supplementary Table 4.** Examination of the interaction between sleep duration and sleep disorder on BMI.

| **Sleep duration** | **Sleep disorder** | **Model 1^a^** | **Model 2^b^** | **Model 3^c^** |
| --- | --- | --- | --- | --- |
| 7−9 h | No | Ref | Ref | Ref |
| < 7 h | No | 0.61 (0.35, 0.87)  < 0.001 | 0.48 (0.23, 0.74)  < 0.001 | 0.37 (0.13, 0.61) 0.003 |
| > 9 h | No | 0.05 (−0.44, 0.55) 0.839 | −0.18 (−0.66, 0.31) 0.480 | −0.35 (−0.81, 0.12) 0.144 |
| 7−9 h | Yes | 1.49 (1.22, 1.75)  < 0.001 | 1.34 (1.08, 1.61)  < 0.001 | 0.75 (0.49, 1.00)  < 0.001 |
| < 7 h | Yes | 2.60 (2.28, 2.93)  < 0.001 | 2.37 (2.05, 2.69)  < 0.001 | 1.48 (1.16, 1.79)  < 0.001 |
| > 9 h | Yes | 1.97 (1.27, 2.67)  < 0.001 | 1.69 (1.00, 2.38)  < 0.001 | 0.73 (0.07, 1.39) 0.031 |
| P for interaction |  | 0.076 | 0.043 | 0.215 |

^a^ Model 1 adjust for: none. ^b^ Model 2 adjust for: age, gender, race. ^c^ Model 3 adjust for: Model 2 plus education level, marital status, poverty income ratio, physical activity, sedentariness, alcohol, smoking, energy intake, hypertension, diabetes, stroke, depression, thyroid disease, cancer or malignancy. BMI, body mass index; h, hour; Ref, reference.

**Supplementary Table 5** Threshold effect analysis of sleep duration on WC.

|  | **Male** |  | **Female** |  |
| --- | --- | --- | --- | --- |
|  | β (95% CI) *P* value | β (95% CI) *P* value | | |
| **One linear model** | 0.07 (−0.04, 0.18) 0.194 | −0.06 (−0.17, 0.05) 0.257 | | |
| **Piecewise model** |  |  | | |
| Inflection point (h) | 8.5 | 9 | | |
| Model 1: sleep duration ≤ inflection point | 0.16 (0.02, 0.29) 0.025 | −0.09 (−0.22, 0.03) 0.151 | | |
| Model 2: sleep duration > inflection point | −0.30 (−0.69, 0.08) 0.123 | 0.12 (−0.30, 0.55) 0.567 | | |
| Model 1 and 2 effect differences | −0.46 (−0.91, −0.01) 0.046 | 0.22 (−0.26, 0.70) 0.371 | | |
| *P* for logarithmic likelihood ratio test | 0.045 | 0.369 | | |

WC, waist circumference; CI, confidence interval; h, hour.

**Supplementary Table 6.** Stratified logistic regression analysis was used to identify variables that affect the correlation between sleep duration and WC.

| **Subgroup** | ***n*** | **7**−**9 h** | **< 7 h** | |  | **> 9 h** | |  |
| --- | --- | --- | --- | --- | --- | --- | --- | --- |
|  |  |  | **β (95% CI)** | ***P* value** | | **β (95% CI)** | ***P* value** | |
| **Age** |  |  |  |  | |  |  | |
| 20−34 | 4681 | Ref | 1.12 (0.03, 2.20) | 0.045 | | −1.04 (−2.95, 0.87) | 0.287 | |
| 35−49 | 5029 | Ref | 0.73 (−0.17, 1.63) | 0.111 | | 2.18 (−0.10, 4.47) | 0.061 | |
| 50−63 | 4954 | Ref | −0.12 (−0.99, 0.75) | 0.788 | | 0.77 (−1.21, 2.75) | 0.446 | |
| ≥ 64 | 5287 | Ref | 0.64 (−0.25, 1.53) | 0.158 | | −0.29 (−1.53, 0.96) | 0.650 | |
| **Gender** |  |  |  |  | |  |  | |
| Male | 9825 | Ref | 0.11 (−0.51, 0.74) | 0.720 | | −0.89 (−2.22, 0.44) | 0.189 | |
| Female | 10126 | Ref | 1.10 (0.41, 1.80) | 0.002 | | 0.61 (−0.61, 1.83) | 0.330 | |
| **Race** |  |  |  |  | |  |  | |
| Mexican American | 2708 | Ref | 1.35 (0.13, 2.58) | 0.030 | | 0.71 (−1.54, 2.96) | 0.536 | |
| Other Hispanic | 2098 | Ref | 0.23 (−1.05, 1.50) | 0.727 | | 0.51 (−2.08, 3.10) | 0.700 | |
| Non-Hispanic White | 7355 | Ref | 0.49 (−0.30, 1.29) | 0.224 | | 0.59 (−0.92, 2.10) | 0.445 | |
| Non-Hispanic Black | 4507 | Ref | 1.14 (0.11, 2.17) | 0.030 | | −0.84 (−2.81, 1.14) | 0.408 | |
| Non-Hispanic Asian | 2574 | Ref | 0.62 (−0.32, 1.56) | 0.198 | | −0.54 (−2.39, 1.31) | 0.566 | |
| Other Race | 709 | Ref | −0.52 (−3.10, 2.07) | 0.696 | | −4.73 (−9.64, 0.17) | 0.059 | |
| **Education** |  |  |  |  | |  |  | |
| Less than 9th grade | 1812 | Ref | 1.15 (−0.23, 2.54) | 0.103 | | 0.53 (−1.65, 2.71) | 0.632 | |
| 9-11th grade | 2514 | Ref | 0.81 (−0.52, 2.13) | 0.233 | | 0.34 (−1.67, 2.35) | 0.742 | |
| High school graduate | 4435 | Ref | 0.40 (−0.62, 1.42) | 0.442 | | −0.25 (−2.05, 1.56) | 0.790 | |
| Some college or AA degree | 6151 | Ref | 0.57 (−0.30, 1.44) | 0.202 | | 0.23 (−1.55, 2.00) | 0.802 | |
| College graduate or above | 5026 | Ref | 0.58 (−0.34, 1.49) | 0.219 | | 0.53 (−1.60, 2.65) | 0.627 | |
| Missing | 13 | Ref | - | - | | - | - | |
| **Marital** |  |  |  |  | |  |  | |
| Married | 10107 | Ref | 0.67 (0.04, 1.30) | 0.038 | | 0.20 (−1.18, 1.58) | 0.775 | |
| Widowed | 1403 | Ref | 0.85 (−0.87, 2.58) | 0.331 | | 0.15 (−2.30, 2.60) | 0.906 | |
| Divorced | 2181 | Ref | −0.46 (−1.81, 0.89) | 0.503 | | −1.45 (−4.03, 1.12) | 0.269 | |
| Separated | 689 | Ref | 1.25 (−1.47, 3.98) | 0.368 | | −2.52 (−7.36, 2.32) | 0.308 | |
| Never married | 3858 | Ref | 1.03 (−0.16, 2.21) | 0.090 | | 0.28 (−1.80, 2.36) | 0.793 | |
| Living with partner | 1704 | Ref | 0.24 (−1.47, 1.95) | 0.785 | | 2.64 (−0.11, 5.38) | 0.060 | |
| Missing | 9 | Ref | - | - | | - | - | |
| **Poverty income ratio** |  |  |  |  | |  |  | |
| ≤ 1.3 | 5867 | Ref | 1.16 (0.26, 2.06) | 0.012 | | 1.27 (−0.14, 2.69) | 0.078 | |
| > 1.3 and ≤ 3.5 | 6745 | Ref | 0.52 (−0.31, 1.34) | 0.219 | | 0.36 (−1.19, 1.91) | 0.650 | |
| > 3.5 | 5471 | Ref | 0.35 (−0.51, 1.20) | 0.428 | | −0.62 (−2.68, 1.45) | 0.559 | |
| Missing | 1868 | Ref | - | - | | - | - | |
| **Alcohol** |  |  |  |  | |  |  | |
| No | 6361 | Ref | 1.06 (0.24, 1.88) | 0.011 | | 0.64 (−0.99, 2.28) | 0.441 | |
| Yes | 6282 | Ref | 0.29 (−0.59, 1.17) | 0.517 | | −0.62 (−2.40, 1.16) | 0.496 | |
| Missing | 7308 | Ref | - | - | | - | - | |
| **Smoking** |  |  |  |  | |  |  | |
| Non-users | 11414 | Ref | 0.83 (0.20, 1.46) | 0.010 | | −0.22 (−1.44, 0.99) | 0.718 | |
| Current smoking | 3912 | Ref | 1.01 (−0.04, 2.06) | 0.060 | | 1.19 (−0.78, 3.16) | 0.236 | |
| Past smoking | 4610 | Ref | −0.01 (−0.99, 0.97) | 0.987 | | 0.65 (−1.21, 2.51) | 0.494 | |
| Missing | 15 | Ref | - | - | | - | - | |
| **Hypertension** |  |  |  |  | |  |  | |
| No | 11359 | Ref | 0.70 (0.08, 1.32) | 0.026 | | 0.18 (−1.07, 1.44) | 0.773 | |
| Yes | 8575 | Ref | 0.28 (−0.44, 1.00) | 0.442 | | 0.28 (−0.99, 1.55) | 0.664 | |
| Missing | 17 | Ref | - | - | | - | - | |
| **Diabetes** |  |  |  |  | |  |  | |
| No | 16234 | Ref | 0.59 (0.07, 1.11) | 0.027 | | −0.12 (−1.15, 0.91) | 0.818 | |
| Yes | 3348 | Ref | 1.26 (0.13, 2.40) | 0.029 | | 1.14 (−0.68, 2.96) | 0.221 | |
| Borderline | 369 | Ref | −1.24 (−4.44, 1.97) | 0.451 | | 2.94 (−4.05, 9.94) | 0.410 | |
| **Depression** |  |  |  |  | |  |  | |
| No | 16521 | Ref | 0.57 (0.06, 1.09) | 0.030 | | 0.18 (−0.85, 1.21) | 0.731 | |
| Yes | 1849 | Ref | 1.08 (−0.49, 2.66) | 0.178 | | 0.38 (−2.16, 2.91) | 0.769 | |
| Missing | 1581 | Ref | - | - | | - | - | |
| **Stroke** |  |  |  |  | |  |  | |
| No | 19219 | Ref | 0.68 (0.20, 1.16) | 0.006 | | 0.22 (−0.72, 1.15) | 0.650 | |
| Yes | 715 | Ref | −1.75 (−4.28, 0.79) | 0.178 | | −1.57 (−5.02, 1.88) | 0.373 | |
| Missing | 17 | Ref | - | - | | - | - | |
| **Thyroid disease** |  |  |  |  | |  |  | |
| No | 17835 | Ref | 0.75 (0.26, 1.25) | 0.003 | | 0.33 (−0.63, 1.29) | 0.504 | |
| Yes | 2074 | Ref | −0.60 (−2.15, 0.96) | 0.451 | | −1.28 (−4.00, 1.43) | 0.355 | |
| Missing | 42 | Ref | - | - | | - | - | |
| **Cancer or malignancy** |  |  |  |  | |  |  | |
| No | 18086 | Ref | 0.81 (0.31, 1.30) | 0.001 | | 0.09 (−0.88, 1.05) | 0.860 | |
| Yes | 1854 | Ref | −1.13 (−2.74, 0.48) | 0.168 | | 0.72 (−1.83, 3.28) | 0.579 | |
| Missing | 11 | Ref | - | - | | - | - | |
| **Physical activity** |  |  |  |  | |  |  | |
| 0.7−4 | 4374 | Ref | −0.24 (−1.30, 0.81) | 0.649 | | −1.13 (−3.10, 0.84) | 0.262 | |
| 4.1−10.7 | 3164 | Ref | 1.03 (−0.17, 2.22) | 0.092 | | 1.80 (−0.61, 4.22) | 0.144 | |
| 10.8−24 | 3774 | Ref | 1.29 (0.24, 2.33) | 0.016 | | 0.33 (−1.73, 2.40) | 0.753 | |
| 24.1−172 | 3673 | Ref | −0.92 (−1.99, 0.15) | 0.093 | | −3.05 (−5.44, −0.66) | 0.013 | |
| Missing | 4966 | Ref | - | - | | - | - | |
| **Sedentary time** |  |  |  |  | |  |  | |
| 0−4 | 7394 | Ref | 0.02 (−0.72, 0.77) | 0.949 | | −0.75 (−2.15, 0.64) | 0.292 | |
| 4.1−6 | 4389 | Ref | 0.94 (−0.08, 1.96) | 0.072 | | 0.07 (−1.79, 1.92) | 0.943 | |
| 6.1−8 | 3829 | Ref | 1.44 (0.38, 2.50) | 0.008 | | 1.73 (−0.21, 3.68) | 0.081 | |
| 8.1−22 | 4223 | Ref | 0.57 (−0.50, 1.65) | 0.296 | | 0.90 (−1.52, 3.32) | 0.468 | |
| Missing | 116 | Ref | - | - | | - | - | |
| **Energy intake (kcal/d)** |  |  |  |  | |  |  | |
| 14−1462.5 | 4633 | Ref | 0.90 (−0.07, 1.87) | 0.068 | | −0.68 (−2.33, 0.98) | 0.424 | |
| 1462.6−1912 | 4645 | Ref | −0.15 (−1.14, 0.83) | 0.759 | | −0.81 (−2.59, 0.96) | 0.369 | |
| 1912.1−2482.1 | 4639 | Ref | 0.81 (−0.15, 1.77) | 0.098 | | 2.59 (0.67, 4.51) | 0.008 | |
| 2482.2−10025 | 4639 | Ref | 1.01 (0.02, 2.00) | 0.046 | | −0.62 (−2.85, 1.61) | 0.588 | |
| Missing | 1395 | Ref | - | - | | - | - | |

The analysis was adjusted by age, gender, race, education level, marital status, poverty income ratio, physical activity, sedentariness, alcohol, smoking, energy intake, hypertension, diabetes, stroke, depression, thyroid disease, cancer or malignancy. WC, waist circumference; h, hour; n, number; CI, confidence interval; Ref, reference; d, day; MET, metabolic equivalent.

**Supplementary Table 7.** Stratified logistic regression analysis was used to identify variables that affect the correlation between sleep disorder and WC.

| **Subgroup** | ***n*** | **Healthy** | **Sleep disorder** | |
| --- | --- | --- | --- | --- |
|  |  |  | **β (95% CI)** | ***P* value** |
| **Age** |  |  |  |  |
| 20−34 | 4681 | Ref | 2.04 (0.80, 3.28) | 0.001 |
| 35−49 | 5029 | Ref | 2.61 (1.62, 3.60) | < 0.001 |
| 50−63 | 4954 | Ref | 2.21 (1.34, 3.08) | < 0.001 |
| ≥ 64 | 5287 | Ref | 1.54 (0.78, 2.29) | < 0.001 |
| **Gender** |  |  |  |  |
| Male | 9825 | Ref | 2.86 (2.18, 3.54) | < 0.001 |
| Female | 10126 | Ref | 1.70 (1.03, 2.37) | < 0.001 |
| **Race** |  |  |  |  |
| Mexican American | 2708 | Ref | 2.50 (1.00, 3.99) | 0.001 |
| Other Hispanic | 2098 | Ref | 2.71 (1.18, 4.24) | < 0.001 |
| Non-Hispanic White | 7355 | Ref | 1.90 (1.15, 2.65) | < 0.001 |
| Non-Hispanic Black | 4507 | Ref | 2.94 (1.74, 4.14) | < 0.001 |
| Non-Hispanic Asian | 2574 | Ref | 1.15 (-0.03, 2.33) | 0.056 |
| Other Race | 709 | Ref | 2.45 (-0.10, 5.01) | 0.060 |
| **Education** |  |  |  |  |
| Less than 9th grade | 1812 | Ref | 3.72 (2.13, 5.31) | < 0.001 |
| 9-11th grade | 2514 | Ref | 3.83 (2.37, 5.29) | < 0.001 |
| High school graduate | 4435 | Ref | 2.91 (1.85, 3.96) | < 0.001 |
| Some college or AA degree | 6151 | Ref | 1.61 (0.71, 2.51) | < 0.001 |
| College graduate or above | 5026 | Ref | 1.58 (0.71, 2.45) | < 0.001 |
| Missing | 13 | Ref | - | - |
| **Marital** |  |  |  |  |
| Married | 10107 | Ref | 1.60 (0.96, 2.24) | < 0.001 |
| Widowed | 1403 | Ref | 0.57 (−0.97, 2.11) | 0.468 |
| Divorced | 2181 | Ref | 3.51 (2.18, 4.83) | < 0.001 |
| Separated | 689 | Ref | 1.74 (−1.16, 4.63) | 0.240 |
| Never married | 3858 | Ref | 4.90 (3.60, 6.20) | < 0.001 |
| Living with partner | 1704 | Ref | 0.49 (−1.34, 2.32) | 0.598 |
| Missing | 9 | Ref | - | - |
| **Poverty income ratio** |  |  |  |  |
| ≤ 1.3 | 5867 | Ref | 2.51 (1.53, 3.48) | < 0.001 |
| > 1.3 and ≤ 3.5 | 6745 | Ref | 2.39 (1.53, 3.25) | < 0.001 |
| > 3.5 | 5471 | Ref | 1.68 (0.85, 2.50) | < 0.001 |
| Missing | 1868 | Ref | - | - |
| **Alcohol** |  |  |  |  |
| No | 6361 | Ref | 2.44 (1.63, 3.25) | < 0.001 |
| Yes | 6282 | Ref | 1.68 (0.77, 2.58) | < 0.001 |
| Missing | 7308 | Ref | - | - |
| **Smoking** |  |  |  |  |
| Non-users | 11414 | Ref | 2.16 (1.50, 2.82) | < 0.001 |
| Current smoking | 3912 | Ref | 1.67 (0.56, 2.77) | 0.003 |
| Past smoking | 4610 | Ref | 2.64 (1.73, 3.55) | < 0.001 |
| Missing | 15 | Ref | - | - |
| **Hypertension** |  |  |  |  |
| No | 11359 | Ref | 0.95 (0.29, 1.62) | 0.005 |
| Yes | 8575 | Ref | 3.50 (2.82, 4.18) | < 0.001 |
| Missing | 17 | Ref | - | - |
| **Diabetes** |  |  |  |  |
| No | 16234 | Ref | 1.75 (1.21, 2.29) | < 0.001 |
| Yes | 3348 | Ref | 4.39 (3.32, 5.46) | < 0.001 |
| Missing | 369 | Ref | - | - |
| **Depression** |  |  |  |  |
| No | 16521 | Ref | 2.11 (1.59, 2.64) | < 0.001 |
| Yes | 1849 | Ref | 2.72 (1.11, 4.34) | 0.001 |
| Missing | 1581 | Ref | - | - |
| **Stroke** |  |  |  |  |
| No | 19219 | Ref | 2.17 (1.68, 2.66) | < 0.001 |
| Yes | 715 | Ref | 2.49 (0.21, 4.78) | 0.033 |
| Missing | 17 | Ref | - | - |
| **Thyroid disease** |  |  |  |  |
| No | 17835 | Ref | 2.35 (1.83, 2.86) | < 0.001 |
| Yes | 2074 | Ref | 1.39 (−0.00, 2.79) | 0.051 |
| Missing | 42 | Ref | - | - |
| **Cancer or malignancy** |  |  |  |  |
| No | 18086 | Ref | 2.32 (1.81, 2.84) | < 0.001 |
| Yes | 1854 | Ref | 1.48 (0.10, 2.85) | 0.035 |
| Missing | 11 | Ref | - | - |
| **Physical activity** |  |  |  |  |
| 0.7-4 | 4374 | Ref | 1.83 (0.81, 2.84) | < 0.001 |
| 4.1-10.7 | 3164 | Ref | 2.62 (1.46, 3.78) | < 0.001 |
| 10.8-24 | 3774 | Ref | 2.21 (1.13, 3.28) | < 0.001 |
| 24.1-172 | 3673 | Ref | 1.98 (0.77, 3.20) | 0.001 |
| Missing | 4966 | Ref | - | - |
| **Sedentary time** |  |  |  |  |
| 0−4 | 7394 | Ref | 1.34 (0.54, 2.15) | 0.001 |
| 4.1−6 | 4389 | Ref | 1.90 (0.87, 2.93) | < 0.001 |
| 6.1−8 | 3829 | Ref | 2.15 (1.09, 3.22) | < 0.001 |
| 8.1−22 | 4223 | Ref | 3.53 (2.48, 4.57) | < 0.001 |
| Missing | 116 | Ref | - | - |
| **Energy intake (kcal/d)** |  |  |  |  |
| 14−1462.5 | 4633 | Ref | 2.13 (1.17, 3.10) | < 0.001 |
| 1462.6−1912 | 4645 | Ref | 1.99 (1.01, 2.97) | < 0.001 |
| 1912.1−2482.1 | 4639 | Ref | 2.29 (1.32, 3.25) | < 0.001 |
| 2482.2−10025 | 4639 | Ref | 1.81 (0.76, 2.87) | < 0.001 |
| Missing | 1395 | Ref | - | - |

The analysis was adjusted by age, gender, race, education level, marital status, poverty income ratio, physical activity, sedentariness, alcohol, smoking, energy intake, hypertension, diabetes, stroke, depression, thyroid disease, cancer or malignancy. WC, waist circumference; n, number; CI, confidence interval; Ref, reference; d, day; MET, metabolic equivalent.

**Supplementary Table 8.** Examination of the interaction between sleep duration and sleep disorder on WC.

| **Sleep duration** | **Sleep disorder** | **Model 1^a^** | **Model 2^b^** | **Model 3^c^** |
| --- | --- | --- | --- | --- |
| 7−9 h | No | Ref | Ref | Ref |
| < 7 h | No | 0.99 (0.37, 1.62) 0.002 | 0.91 (0.30, 1.51) 0.004 | 0.58 (0.01, 1.15) 0.047 |
| > 9 h | No | 0.62 (−0.57, 1.82) 0.307 | 0.32 (−0.83, 1.47) 0.585 | −0.22 (−1.31, 0.86) 0.687 |
| 7−9 h | Yes | 4.61 (3.97, 5.26)  < 0.001 | 3.79 (3.16, 4.42)  < 0.001 | 2.19 (1.60, 2.79)  < 0.001 |
| < 7 h | Yes | 6.42 (5.64, 7.20)  < 0.001 | 5.51 (4.76, 6.26)  < 0.001 | 3.16 (2.43, 3.90)  < 0.001 |
| > 9 h | Yes | 5.86 (4.16, 7.55)  < 0.001 | 5.00 (3.37, 6.63)  < 0.001 | 2.30 (0.75, 3.85) 0.004 |
| P for interaction | | 0.253 | 0.329 | 0.265 |

^a^ Model 1 adjust for: none. ^b^ Model 2 adjust for: age, gender, race. ^c^ Model 3 adjust for: Model 2 plus education level, marital status, poverty income ratio, physical activity, sedentariness, alcohol, smoking, energy intake, hypertension, diabetes, stroke, depression, thyroid disease, cancer or malignancy. WC, waist circumference; h, hour; Ref, reference.

**Supplementary Table 9.** Threshold effect analysis of sleep duration on lean mass.

|  | **Male** |  | **Female** |  | |
| --- | --- | --- | --- | --- | --- |
|  | **β (95% CI) *P* value** | **β (95% CI) *P* value** | | |  |
| **One linear model** | −0.32 (−0.51, −0.13) 0.001 | −0.18 (−0.34, −0.03) 0.020 | | |  |
| **Piecewise model** |  |  | | |  |
| Inflection point (h) | 8.5 | 5 | | |  |
| Model 1: sleep duration ≤ inflection point | −0.26 (−0.50, −0.03) 0.028 | 0.58 (−0.38, 1.54) 0.239 | | |  |
| Model 2: sleep duration > inflection point | −0.56 (−1.22, 0.10) 0.097 | −0.24 (−0.41, −0.07) 0.005 | | |  |
| Model 1 and 2 effect differences | −0.30 (−1.07, 0.48) 0.453 | −0.82 (−1.84, 0.20) 0.117 | | |  |
| *P* for logarithmic likelihood ratio test | 0.451 | 0.115 | | |  |

h, hour; CI, confidence interval

**Supplementary Table 10.** Stratified logistic regression analysis was used to identify variables that affect the correlation between sleep duration and lean mass.

| **Subgroup** | ***n*** | **7**−**9 h** | **< 7 h** | |  | **> 9 h** | |  |
| --- | --- | --- | --- | --- | --- | --- | --- | --- |
|  |  |  | **β (95% CI)** | ***P* value** | | **β (95% CI)** | ***P* value** | |
| **Age** |  |  |  |  | |  |  | |
| 20−29 | 2441 | Ref | 0.61 (−0.26, 1.48) | 0.172 | | −1.13 (−2.61, 0.34) | 0.132 | |
| 30−39 | 2686 | Ref | 0.28 (−0.50, 1.05) | 0.484 | | −0.25 (−1.84, 1.34) | 0.761 | |
| 40−49 | 2720 | Ref | 0.58 (−0.11, 1.27) | 0.101 | | −0.49 (−2.44, 1.46) | 0.623 | |
| 50−59 | 2869 | Ref | 0.66 (0.00, 1.32) | 0.049 | | −0.93 (−2.59, 0.73) | 0.271 | |
| **Gender** |  |  |  |  | |  |  | |
| Male | 5347 | Ref | 0.56 (0.01, 1.11) | 0.047 | | −1.28 (−2.59, 0.03) | 0.056 | |
| Female | 5369 | Ref | 0.57 (0.08, 1.06) | 0.024 | | −0.22 (−1.21, 0.76) | 0.657 | |
| **Race** |  |  |  |  | |  |  | |
| Mexican American | 1602 | Ref | 0.96 (0.01, 1.91) | 0.049 | | −2.29 (−4.23, −0.34) | 0.021 | |
| Other Hispanic | 1126 | Ref | 0.91 (−0.19, 2.02) | 0.106 | | 0.79 (−1.62, 3.20) | 0.521 | |
| Non-Hispanic White | 3708 | Ref | 0.59 (−0.04, 1.22) | 0.067 | | −0.02 (−1.44, 1.40) | 0.980 | |
| Non-Hispanic Black | 2243 | Ref | 0.41 (−0.46, 1.28) | 0.359 | | −1.35 (−3.18, 0.49) | 0.151 | |
| Non-Hispanic Asian | 1598 | Ref | 0.95 (0.19, 1.72) | 0.015 | | −1.09 (−2.68, 0.50) | 0.180 | |
| Other Race | 439 | Ref | −0.07 (−2.09, 1.96) | 0.948 | | −3.22 (−7.26, 0.81) | 0.119 | |
| **Education level** |  |  |  |  | |  |  | |
| Less than 9th grade | 680 | Ref | 0.28 (−1.05, 1.62) | 0.678 | | 0.42 (−2.42, 3.27) | 0.770 | |
| 9-11th grade | 1282 | Ref | 1.17 (0.04, 2.29) | 0.042 | | 0.75 (−1.13, 2.63) | 0.435 | |
| High school graduate | 2335 | Ref | 0.21 (−0.59, 1.01) | 0.608 | | −0.13 (−1.64, 1.39) | 0.871 | |
| Some college or AA degree | 3488 | Ref | 0.92 (0.25, 1.59) | 0.007 | | −2.47 (−4.00, −0.93) | 0.002 | |
| College graduate or above | 2929 | Ref | 0.41 (−0.28, 1.09) | 0.245 | | 0.61 (−1.33, 2.55) | 0.538 | |
| Missing | 2 | Ref | - | - | |  |  | |
| **Marital status** |  |  |  |  | |  |  | |
| Married | 5231 | Ref | 0.53 (0.02, 1.04) | 0.041 | | −1.19 (−2.60, 0.21) | 0.097 | |
| Widowed | 145 | Ref | 0.10 (−2.86, 3.06) | 0.946 | | −3.67 (−13.47, 6.13) | 0.464 | |
| Divorced | 961 | Ref | 0.60 (−0.51, 1.72) | 0.289 | | −1.54 (−4.00, 0.92) | 0.221 | |
| Separated | 381 | Ref | 2.03 (−0.06, 4.12) | 0.058 | | 1.17 (−2.83, 5.16) | 0.567 | |
| Never married | 2793 | Ref | 0.36 (−0.45, 1.16) | 0.383 | | −0.20 (−1.63, 1.22) | 0.782 | |
| Living with partner | 1204 | Ref | 0.99 (−0.21, 2.20) | 0.106 | | 0.60 (−1.37, 2.58) | 0.550 | |
| Missing | 1 | Ref | - | - | | - | - | |
| **Poverty income ratio** |  |  |  |  | |  |  | |
| ≤ 1.3 | 3211 | Ref | 0.47 (−0.23, 1.16) | 0.191 | | −0.14 (−1.29, 1.02) | 0.818 | |
| > 1.3 and ≤ 3.5 | 3532 | Ref | 0.65 (−0.01, 1.31) | 0.054 | | −1.53 (−3.02, −0.05) | 0.043 | |
| > 3.5 | 3115 | Ref | 0.62 (−0.03, 1.28) | 0.063 | | 0.36 (−1.65, 2.36) | 0.727 | |
| Missing | 858 | Ref | - | - | | - | - | |
| **Alcohol** |  |  |  |  | |  |  | |
| No | 3279 | Ref | 1.09 (0.42, 1.75) | 0.002 | | −0.46 (−2.15, 1.23) | 0.592 | |
| Yes | 4292 | Ref | 0.61 (0.03, 1.20) | 0.039 | | −0.80 (−2.08, 0.47) | 0.217 | |
| Missing | 3145 | Ref | - | - | | - | - | |
| **Smoking** |  |  |  |  | |  |  | |
| Non-users | 6517 | Ref | 0.59 (0.11, 1.07) | 0.016 | | −0.74 (−1.81, 0.33) | 0.175 | |
| Current smoking | 2415 | Ref | 0.92 (0.16, 1.69) | 0.018 | | −0.15 (−1.59, 1.28) | 0.833 | |
| Past smoking | 1777 | Ref | 0.37 (−0.57, 1.31) | 0.439 | | −0.53 (−2.87, 1.82) | 0.660 | |
| Missing | 7 | Ref | - | - | | - | - | |
| **Hypertension** |  |  |  |  | |  |  | |
| No | 7715 | Ref | 0.68 (0.26, 1.11) | 0.002 | | −0.35 (−1.27, 0.57) | 0.455 | |
| Yes | 2994 | Ref | 0.42 (−0.31, 1.15) | 0.260 | | −0.80 (−2.44, 0.85) | 0.342 | |
| Missing | 7 | Ref | - | - | | - | - | |
| **Diabetes** |  |  |  |  | |  |  | |
| No | 9538 | Ref | 0.58 (0.20, 0.97) | 0.003 | | −0.39 (−1.23, 0.46) | 0.368 | |
| Yes | 1034 | Ref | 0.65 (−0.71, 2.02) | 0.349 | | −1.82 (−4.77, 1.13) | 0.228 | |
| Borderline | 144 | Ref | 1.39 (−2.72, 5.50) | 0.508 | | −6.59 (−14.88, 1.70) | 0.123 | |
| **Depression** |  |  |  |  | |  |  | |
| No | 9058 | Ref | 0.64 (0.24, 1.04) | 0.002 | | −0.74 (−1.67, 0.19) | 0.119 | |
| Yes | 955 | Ref | 1.19 (−0.06, 2.44) | 0.062 | | 0.25 (−1.87, 2.38) | 0.816 | |
| Missing | 703 | Ref | - | - | | - | - | |
| **Stroke** |  |  |  |  | |  |  | |
| No | 10558 | Ref | 0.62 (0.25, 1.00) | 0.001 | | −0.62 (−1.44, 0.19) | 0.134 | |
| Yes | 154 | Ref | 1.78 (−1.55, 5.12) | 0.298 | | 2.39 (−4.22, 9.00) | 0.480 | |
| Missing | 4 | Ref | - | - | | - | - | |
| **Thyroid disease** |  |  |  |  | |  |  | |
| No | 9980 | Ref | 0.75 (0.37, 1.13) | < 0.001 | | −0.64 (−1.48, 0.21) | 0.138 | |
| Yes | 715 | Ref | −1.24 (−2.73, 0.26) | 0.106 | | 0.19 (−2.81, 3.19) | 0.902 | |
| Missing | 21 | Ref | - | - | | - | - | |
| **Cancer or malignancy** |  |  |  |  | |  |  | |
| No | 10310 | Ref | 0.55 (0.17, 0.92) | 0.005 | | −0.62 (−1.44, 0.21) | 0.142 | |
| Yes | 404 | Ref | 1.34 (−0.61, 3.28) | 0.179 | | 0.25 (−4.19, 4.68) | 0.914 | |
| Missing | 2 | Ref | - | - | | - | - | |
| **Physical activity (MET-h)** |  |  |  |  | |  |  | |
| 0.7−4 | 2089 | Ref | −0.23 (−1.04, 0.58) | 0.575 | | −1.44 (−3.26, 0.39) | 0.123 | |
| 4.1−10.7 | 1800 | Ref | 0.85 (−0.07, 1.78) | 0.072 | | 1.52 (−0.47, 3.51) | 0.134 | |
| 10.8−24 | 2282 | Ref | 0.92 (0.12, 1.72) | 0.025 | | −0.22 (−2.00, 1.56) | 0.807 | |
| 24.1−172 | 2476 | Ref | −0.29 (−1.07, 0.48) | 0.460 | | −3.31 (−5.13, −1.49) | < 0.001 | |
| Missing | 2069 | Ref | - | - | | - | - | |
| **Sedentary time (h)** |  |  |  |  | |  |  | |
| 0−4 | 4006 | Ref | 0.04 (−0.55, 0.63) | 0.900 | | −1.41 (−2.65, −0.17) | 0.026 | |
| 4.1−6 | 2220 | Ref | 1.38 (0.58, 2.17) | < 0.001 | | 0.32 (−1.38, 2.03) | 0.710 | |
| 6.1−8 | 2035 | Ref | 0.93 (0.07, 1.80) | 0.035 | | −0.08 (−1.82, 1.65) | 0.925 | |
| 8.1−22 | 2411 | Ref | 0.44 (−0.38, 1.26) | 0.296 | | −0.52 (−2.69, 1.64) | 0.636 | |
| Missing | 44 | Ref | - | - | | - | - | |
| **Energy intake (kcal/d)** |  |  |  |  | |  |  | |
| 14−1545 | 2542 | Ref | −0.36 (−1.12, 0.40) | 0.350 | | −1.48 (−2.93, −0.02) | 0.046 | |
| 1545.1−2010 | 2542 | Ref | 0.57 (−0.18, 1.32) | 0.134 | | −0.78 (−2.41, 0.86) | 0.352 | |
| 2010.1−2603 | 2541 | Ref | 1.27 (0.52, 2.03) | 0.001 | | 0.71 (−1.09, 2.51) | 0.439 | |
| 2603.1−9595 | 2544 | Ref | 0.80 (0.02, 1.59) | 0.045 | | −1.40 (−3.26, 0.46) | 0.139 | |
| Missing | 547 | Ref | - | - | | - | - | |

The analysis was adjusted by age, gender, race, education level, marital status, poverty income ratio, physical activity, sedentariness, alcohol, smoking, energy intake, hypertension, diabetes, stroke, depression, thyroid disease, cancer or malignancy. h, hour; n, number; CI, confidence interval; Ref, reference; d, day; MET, metabolic equivalent.

**Supplementary Table 11.** Stratified logistic regression analysis was used to identify variables that affect the correlation between sleep disorder and lean mass.

| **Subgroup** | ***n*** | **Healthy** | **Sleep disorder** | |
| --- | --- | --- | --- | --- |
|  |  |  | **β (95% CI)** | ***P* value** |
| **Age** |  |  |  |  |
| 20−29 | 2441 | Ref | −0.55 (−1.59, 0.50) | 0.304 |
| 30−39 | 2686 | Ref | 0.22 (−0.67, 1.12) | 0.625 |
| 40−49 | 2720 | Ref | 1.16 (0.41, 1.90) | 0.002 |
| 50−59 | 2869 | Ref | 0.97 (0.30, 1.64) | 0.005 |
| **Gender** |  |  |  |  |
| Male | 5347 | Ref | 0.50 (−0.14, 1.15) | 0.125 |
| Female | 5369 | Ref | 0.69 (0.18, 1.19) | 0.008 |
| **Race** |  |  |  |  |
| Mexican American | 1602 | Ref | 1.55 (0.31, 2.79) | 0.014 |
| Other Hispanic | 1126 | Ref | 1.07 (−0.35, 2.50) | 0.141 |
| Non-Hispanic White | 3708 | Ref | 0.35 (−0.30, 1.00) | 0.290 |
| Non-Hispanic Black | 2243 | Ref | 1.28 (0.21, 2.35) | 0.019 |
| Non-Hispanic Asian | 1598 | Ref | 0.49 (−0.53, 1.50) | 0.347 |
| Other Race | 439 | Ref | 1.26 (−0.92, 3.43) | 0.259 |
| **Education level** |  |  |  |  |
| Less than 9th grade | 680 | Ref | 1.26 (−0.53, 3.05) | 0.167 |
| 9-11th grade | 1282 | Ref | 1.60 (0.24, 2.96) | 0.021 |
| High school graduate | 2335 | Ref | 1.72 (0.82, 2.62) | < 0.001 |
| Some college or AA degree | 3488 | Ref | 0.26 (−0.48, 1.00) | 0.496 |
| College graduate or above | 2929 | Ref | 0.00 (−0.70, 0.70) | 0.997 |
| Missing | 2 | Ref | - | - |
| **Marital status** |  |  |  |  |
| Married | 5231 | Ref | 0.92 (0.35, 1.48) | 0.002 |
| Widowed | 145 | Ref | −1.73 (−4.97, 1.50) | 0.297 |
| Divorced | 961 | Ref | 0.49 (−0.63, 1.62) | 0.390 |
| Separated | 381 | Ref | 2.51 (0.22, 4.81) | 0.033 |
| Never married | 2793 | Ref | 0.21 (−0.71, 1.13) | 0.660 |
| Living with partner | 1204 | Ref | 0.59 (−0.69, 1.87) | 0.366 |
| Missing | 1 | Ref | - | - |
| **Poverty income ratio** |  |  |  |  |
| ≤ 1.3 | 3211 | Ref | 0.50 (−0.30, 1.30) | 0.223 |
| > 1.3 and ≤ 3.5 | 3532 | Ref | 0.86 (0.10, 1.62) | 0.027 |
| > 3.5 | 3115 | Ref | 0.32 (−0.36, 1.01) | 0.353 |
| Missing | 858 | Ref | - | - |
| **Alcohol** |  |  |  |  |
| No | 3279 | Ref | 0.92 (0.19, 1.64) | 0.013 |
| Yes | 4292 | Ref | 0.03 (−0.61, 0.67) | 0.920 |
| Missing | 3145 | Ref | - | - |
| **Smoking** |  |  |  |  |
| Non-users | 6517 | Ref | 0.89 (0.35, 1.43) | 0.001 |
| Current smoking | 2415 | Ref | 0.16 (−0.66, 0.98) | 0.705 |
| Past smoking | 1777 | Ref | 0.60 (−0.36, 1.56) | 0.220 |
| Missing | 7 | Ref | - | - |
| **Hypertension** |  |  |  |  |
| No | 7715 | Ref | 0.08 (−0.41, 0.57) | 0.740 |
| Yes | 2994 | Ref | 1.75 (1.01, 2.49) | <0.001 |
| Missing | 7 | Ref | - | - |
| **Diabetes** |  |  |  |  |
| No | 9538 | Ref | 0.49 (0.06, 0.92) | 0.025 |
| Yes | 1034 | Ref | 1.39 (−0.02, 2.80) | 0.054 |
| Borderline | 144 | Ref | 4.21 (−0.04, 8.47) | 0.055 |
| **Depression** |  |  |  |  |
| No | 9058 | Ref | 0.57 (0.12, 1.02) | 0.013 |
| Yes | 955 | Ref | 1.07 (−0.23, 2.36) | 0.106 |
| Missing | 703 | Ref | - | - |
| **Stroke** |  |  |  |  |
| No | 10558 | Ref | 0.61 (0.19, 1.02) | 0.004 |
| Yes | 154 | Ref | 0.84 (−2.64, 4.31) | 0.638 |
| Missing | 4 | Ref | - | - |
| **Thyroid disease** |  |  |  |  |
| No | 9980 | Ref | 0.58 (0.15, 1.01) | 0.008 |
| Yes | 715 | Ref | 1.12 (−0.34, 2.58) | 0.134 |
| Missing | 21 | Ref | - | - |
| **Cancer or malignancy** |  |  |  |  |
| No | 10310 | Ref | 0.61 (0.19, 1.03) | 0.004 |
| Yes | 404 | Ref | 1.18 (−0.66, 3.02) | 0.209 |
| Missing | 2 | Ref | - | - |
| **Physical activity (MET-h)** |  |  |  |  |
| 0.7−4 | 2089 | Ref | 0.94 (0.07, 1.81) | 0.034 |
| 4.1−10.7 | 1800 | Ref | 0.52 (−0.43, 1.48) | 0.284 |
| 10.8−24 | 2282 | Ref | 0.88 (−0.00, 1.75) | 0.051 |
| 24.1−172 | 2476 | Ref | −0.32 (−1.26, 0.62) | 0.506 |
| Missing | 2069 | Ref | - | - |
| **Sedentary time (h)** |  |  |  |  |
| 0−4 | 4006 | Ref | 0.95 (0.24, 1.65) | 0.008 |
| 4.1−6 | 2220 | Ref | −0.02 (−0.93, 0.88) | 0.958 |
| 6.1−8 | 2035 | Ref | 0.63 (−0.30, 1.57) | 0.184 |
| 8.1−22 | 2411 | Ref | 0.86 (0.03, 1.68) | 0.042 |
| Missing | 44 | Ref | - | - |
| **Energy intake (kcal/d)** |  |  |  |  |
| 14−1545 | 2542 | Ref | 0.68 (−0.11, 1.47) | 0.092 |
| 1545.1−2010 | 2542 | Ref | 0.67 (−0.16, 1.50) | 0.112 |
| 2010.1−2603 | 2541 | Ref | 0.24 (−0.59, 1.06) | 0.576 |
| 2603.1−9595 | 2544 | Ref | 0.58 (−0.34, 1.49) | 0.216 |
| Missing | 547 | Ref | - | - |

The analysis was adjusted by age, gender, race, education level, marital status, poverty income ratio, physical activity, sedentariness, alcohol, smoking, energy intake, hypertension, diabetes, stroke, depression, thyroid disease, cancer or malignancy. n, number; CI, confidence interval; Ref, reference; d, day; MET, metabolic equivalent.

**Supplementary Table 12.** Examination of the interaction between sleep duration and sleep disorder on lean mass.

| **Sleep duration** | **Sleep disorder** | **Model 1^a^** | **Model 2^b^** | **Model 3^c^** |
| --- | --- | --- | --- | --- |
| 7−9 h | No | Ref | Ref | Ref |
| < 7 h | No | 2.26 (1.62, 2.90)  < 0.001 | 0.70 (0.24, 1.16) 0.003 | 0.09 (−0.19, 0.38) 0.518 |
| > 9 h | No | −1.46 (−2.85, −0.07) 0.040 | −0.72 (−1.72, 0.28) 0.160 | 0.12 (−0.50, 0.74) 0.700 |
| 7−9 h | Yes | 0.69 (−0.04, 1.41) 0.064 | 1.54 (1.02, 2.07)  < 0.001 | 0.75 (0.42, 1.09)  < 0.001 |
| < 7 h | Yes | 3.10 (2.27, 3.93)  < 0.001 | 2.27 (1.67, 2.88)  < 0.001 | 0.64 (0.25,1.02) 0.001 |
| > 9 h | Yes | −1.46 (−3.52, 0.60) 0.166 | −0.40 (−1.88, 1.08) 0.598 | −0.00 (−0.93, 0.92) 0.992 |
| P for interaction | | 0.253 | 0.818 | 0.402 |

^a^ Model 1 adjust for: none. ^b^ Model 2 adjust for: age, gender, race. ^c^ Model 3 adjust for: Model 2 plus education level, marital status, poverty income ratio, physical activity, sedentariness, alcohol, smoking, energy intake, hypertension, diabetes, stroke, depression, thyroid disease, cancer or malignancy. h, hour; Ref, reference.

**Supplementary Table 13.** Threshold effect analysis of sleep duration on body fat percentage.

|  | **Male** |  | **Female** |  |
| --- | --- | --- | --- | --- |
|  | **β (95% CI) *P* value** | **β (95% CI) *P* value** | | |
| **One linear model** | 0.07 (−0.04, 0.18) 0.194 | −0.06 (−0.17, 0.05) 0.257 | | |
| **Piecewise model** |  |  | | |
| Inflection point (h) | 8.5 | 9 | | |
| Model 1: sleep duration ≤ inflection point | 0.16 (0.02, 0.29) 0.025 | −0.09 (−0.22, 0.03) 0.151 | | |
| Model 2: sleep duration > inflection point | −0.30 (−0.69, 0.08) 0.123 | 0.12 (−0.30, 0.55) 0.567 | | |
| Model 1 and 2 effect differences | −0.46 (−0.91, -0.01) 0.046 | 0.22 (−0.26, 0.70) 0.371 | | |
| *P* for logarithmic likelihood ratio test | 0.045 | 0.369 | | |

h, hour; CI, confidence interval.

**Supplementary Table 14.** Stratified logistic regression analysis was used to identify variables that affect the correlation between sleep duration and body fat percentage.

| **Subgroup** | ***n*** | **7−9 h** | **< 7 h** | |  | **> 9 h** | |  |
| --- | --- | --- | --- | --- | --- | --- | --- | --- |
|  |  |  | **β (95% CI)** | ***P* value** | | **β (95% CI)** | ***P* value** | |
| **Age** |  |  |  |  | |  |  | |
| 20−29 | 2441 | Ref | 0.47 (−0.13, 1.07) | 0.125 | | −0.75 (−1.77, 0.26) | 0.147 | |
| 30−39 | 2686 | Ref | 0.02 (−0.47, 0.51) | 0.947 | | 0.52 (−0.49, 1.53) | 0.312 | |
| 40−49 | 2720 | Ref | −0.03 (−0.46, 0.39) | 0.877 | | 1.45 (0.25, 2.65) | 0.018 | |
| 50−59 | 2869 | Ref | −0.19 (−0.61, 0.22) | 0.359 | | −0.72 (−1.77, 0.32) | 0.174 | |
| **Gender** |  |  |  |  | |  |  | |
| Male | 5347 | Ref | −0.17 (−0.49, 0.15) | 0.289 | | −0.31 (−1.07, 0.45) | 0.418 | |
| Female | 5369 | Ref | 0.26 (−0.10, 0.61) | 0.159 | | −0.08 (−0.79, 0.63) | 0.823 | |
| **Race** |  |  |  |  | |  |  | |
| Mexican American | 1602 | Ref | 0.52 (−0.05, 1.08) | 0.072 | | 0.28 (−0.87, 1.43) | 0.632 | |
| Other Hispanic | 1126 | Ref | −0.08 (−0.76, 0.61) | 0.831 | | 0.82 (−0.69, 2.32) | 0.287 | |
| Non-Hispanic White | 3708 | Ref | 0.08 (−0.34, 0.49) | 0.719 | | −0.06 (−1.00, 0.87) | 0.896 | |
| Non-Hispanic Black | 2243 | Ref | −0.01 (−0.54, 0.52) | 0.969 | | −0.83 (−1.96, 0.29) | 0.148 | |
| Non-Hispanic Asian | 1598 | Ref | −0.20 (−0.77, 0.38) | 0.501 | | 0.21 (−0.98, 1.40) | 0.731 | |
| Other Race | 439 | Ref | −1.06 (−2.28, 0.16) | 0.090 | | −2.83 (−5.26, −0.41) | 0.023 | |
| **Education level** |  |  |  |  | |  |  | |
| Less than 9th grade | 680 | Ref | 0.44 (−0.41, 1.29) | 0.314 | | 1.68 (−0.13, 3.49) | 0.069 | |
| 9-11th grade | 1282 | Ref | −0.02 (−0.68, 0.64) | 0.952 | | −0.02 (−1.12, 1.07) | 0.966 | |
| High school graduate | 2335 | Ref | 0.22 (−0.30, 0.73) | 0.414 | | 0.13 (−0.84, 1.11) | 0.791 | |
| Some college or AA degree | 3488 | Ref | −0.15 (−0.56, 0.26) | 0.467 | | −0.75 (−1.68, 0.19) | 0.117 | |
| College graduate or above | 2929 | Ref | −0.08 (−0.57, 0.40) | 0.745 | | 0.09 (−1.29, 1.46) | 0.903 | |
| Missing | 2 | Ref | - | - | | - | - | |
| **Marital status** |  |  |  |  | |  |  | |
| Married | 5231 | Ref | 0.09 (−0.23, 0.40) | 0.586 | | 1.00 (0.12, 1.87) | 0.026 | |
| Widowed | 145 | Ref | −0.95 (−3.16, 1.26) | 0.400 | | 4.50 (−2.81, 11.82) | 0.230 | |
| Divorced | 961 | Ref | 0.19 (−0.56, 0.93) | 0.624 | | 0.20 (−1.44, 1.85) | 0.808 | |
| Separated | 381 | Ref | −0.93 (−2.20, 0.34) | 0.151 | | −3.78 (−6.20, −1.36) | 0.002 | |
| Never married | 2793 | Ref | 0.05 (−0.48, 0.59) | 0.848 | | −0.96 (−1.91, −0.01) | 0.048 | |
| Living with partner | 1204 | Ref | 0.23 (−0.57, 1.03) | 0.577 | | 0.72 (−0.60, 2.04) | 0.287 | |
| Missing | 1 | Ref | - | - | | - | - | |
| **Poverty income ratio** |  |  |  |  | |  |  | |
| ≤ 1.3 | 3211 | Ref | 0.12 (−0.34, 0.57) | 0.613 | | −0.02 (−0.77, 0.73) | 0.961 | |
| > 1.3 and ≤ 3.5 | 3532 | Ref | 0.26 (−0.15, 0.68) | 0.211 | | 0.26 (−0.67, 1.18) | 0.589 | |
| > 3.5 | 3115 | Ref | −0.28 (−0.71, 0.16) | 0.216 | | 0.06 (−1.27, 1.39) | 0.930 | |
| Missing | 858 | Ref | - | - | | - | - | |
| **Alcohol** |  |  |  |  | |  |  | |
| No | 3279 | Ref | 0.33 (−0.09, 0.75) | 0.128 | | −0.33 (−1.39, 0.74) | 0.548 | |
| Yes | 4292 | Ref | −0.13 (−0.53, 0.26) | 0.500 | | −0.10 (−0.96, 0.75) | 0.817 | |
| Missing | 3145 | Ref | - | - | | - | - | |
| **Smoking** |  |  |  |  | |  |  | |
| Non-users | 6517 | Ref | −0.11 (−0.42, 0.20) | 0.491 | | −0.54 (−1.24, 0.16) | 0.129 | |
| Current smoking | 2415 | Ref | 0.35 (−0.16, 0.86) | 0.178 | | 0.43 (−0.52, 1.39) | 0.375 | |
| Past smoking | 1777 | Ref | −0.02 (−0.58, 0.54) | 0.940 | | 0.53 (−0.85, 1.92) | 0.451 | |
| Missing | 7 | Ref | - | - | | - | - | |
| **Hypertension** |  |  |  |  | |  |  | |
| No | 7715 | Ref | 0.10 (−0.19, 0.39) | 0.512 | | −0.15 (−0.78, 0.47) | 0.633 | |
| Yes | 2994 | Ref | −0.13 (−0.54, 0.29) | 0.548 | | 0.21 (−0.72, 1.14) | 0.659 | |
| Missing | 7 | Ref | - | - | | - | - | |
| **Diabetes** |  |  |  |  | |  |  | |
| No | 9538 | Ref | 0.01 (−0.25, 0.27) | 0.933 | | −0.27 (−0.83, 0.29) | 0.350 | |
| Yes | 1034 | Ref | 0.36 (−0.28, 1.01) | 0.270 | | 0.07 (−1.33, 1.47) | 0.922 | |
| Borderline | 144 | Ref | 0.58 (−1.52, 2.69) | 0.590 | | 6.42 (2.18, 10.67) | 0.004 | |
| **Depression** |  |  |  |  | |  |  | |
| No | 9058 | Ref | 0.00 (−0.26, 0.26) | 0.980 | | −0.39 (−0.99, 0.21) | 0.199 | |
| Yes | 955 | Ref | 0.01 (−0.79, 0.81) | 0.981 | | −0.06 (−1.42, 1.30) | 0.934 | |
| Missing | 703 | Ref | - | - | | - | - | |
| **Stroke** |  |  |  |  | |  |  | |
| No | 10558 | Ref | 0.04 (−0.20, 0.28) | 0.754 | | −0.14 (−0.66, 0.38) | 0.601 | |
| Yes | 154 | Ref | 0.36 (−1.91, 2.63) | 0.756 | | −0.60 (−5.10, 3.89) | 0.793 | |
| Missing | 4 | Ref | - | - | | - | - | |
| **Thyroid disease** |  |  |  |  | |  |  | |
| No | 9980 | Ref | 0.08 (−0.17, 0.33) | 0.542 | | −0.21 (−0.75, 0.34) | 0.460 | |
| Yes | 715 | Ref | −0.49 (−1.41, 0.43) | 0.299 | | 0.94 (−0.90, 2.79) | 0.317 | |
| Missing | 21 | Ref | - | - | | - | - | |
| **Cancer or malignancy** |  |  |  |  | |  |  | |
| No | 10310 | Ref | 0.10 (−0.15, 0.34) | 0.434 | | −0.11 (−0.64, 0.43) | 0.698 | |
| Yes | 404 | Ref | −0.70 (−1.93, 0.53) | 0.267 | | 0.41 (−2.40, 3.22) | 0.776 | |
| Missing | 2 | Ref | - | - | | - | - | |
| **Physical activity (MET-h)** |  |  |  |  | |  |  | |
| 0.7−4 | 2089 | Ref | 0.12 (−0.41, 0.66) | 0.650 | | −1.04 (−2.24, 0.15) | 0.087 | |
| 4.1−10.7 | 1800 | Ref | −0.49 (−1.11, 0.14) | 0.130 | | 0.06 (−1.29, 1.41) | 0.931 | |
| 10.8−24 | 2282 | Ref | 0.21 (−0.32, 0.73) | 0.443 | | −0.26 (−1.42, 0.90) | 0.658 | |
| 24.1−172 | 2476 | Ref | −0.18 (−0.69, 0.32) | 0.477 | | −0.55 (−1.74, 0.63) | 0.362 | |
| Missing | 2069 | Ref | - | - | | - | - | |
| **Sedentary time (h)** |  |  |  |  | |  |  | |
| 0−4 | 4006 | Ref | 0.09 (−0.31, 0.48) | 0.662 | | −0.59 (−1.42, 0.24) | 0.162 | |
| 4.1−6 | 2220 | Ref | 0.53 (0.01, 1.06) | 0.046 | | 0.83 (−0.29, 1.95) | 0.149 | |
| 6.1−8 | 2035 | Ref | 0.32 (−0.23, 0.86) | 0.253 | | −0.10 (−1.19, 0.99) | 0.855 | |
| 8.1−22 | 2411 | Ref | −0.66 (−1.17, −0.15) | 0.011 | | −0.19 (−1.53, 1.16) | 0.782 | |
| Missing | 44 | Ref | - | - | | - | - | |
| **Energy intake (kcal/d)** |  |  |  |  | |  |  | |
| 14−1545 | 2542 | Ref | 0.33 (−0.19, 0.84) | 0.212 | | −0.31 (−1.29, 0.67) | 0.533 | |
| 1545.1−2010 | 2542 | Ref | −0.14 (−0.62, 0.35) | 0.584 | | −0.84 (−1.91, 0.22) | 0.122 | |
| 2010.1−2603 | 2541 | Ref | −0.15 (−0.65, 0.34) | 0.545 | | 0.42 (−0.75, 1.60) | 0.480 | |
| 2603.1−9595 | 2544 | Ref | 0.21 (−0.28, 0.69) | 0.402 | | 0.34 (−0.79, 1.48) | 0.554 | |
| Missing | 547 | Ref | - | - | | - | - | |

The analysis was adjusted by age, gender, race, education level, marital status, poverty income ratio, physical activity, sedentariness, alcohol, smoking, energy intake, hypertension, diabetes, stroke, depression, thyroid disease, cancer or malignancy. h, hour; n, number; CI, confidence interval; Ref, reference; d, day; MET, metabolic equivalent.

**Supplementary Table 15.** Stratified logistic regression analysis was used to identify variables that affect the correlation between sleep disorder and body fat percentage.

| **Subgroup** | ***n*** | **Healthy** | **Sleep disorder** | |
| --- | --- | --- | --- | --- |
|  |  |  | **β (95% CI)** | ***P* value** |
| **Age** |  |  |  |  |
| 20−29 | 2441 | Ref | 0.42 (−0.30, 1.14) | 0.255 |
| 30−39 | 2686 | Ref | 0.61 (0.05, 1.18) | 0.034 |
| 40−49 | 2720 | Ref | 0.31 (−0.15, 0.77) | 0.181 |
| 50−59 | 2869 | Ref | 0.85 (0.43, 1.28) | <0.001 |
| **Gender** |  |  |  |  |
| Male | 5347 | Ref | 0.90 (0.53, 1.27) | <0.001 |
| Female | 5369 | Ref | 0.28 (−0.09, 0.64) | 0.142 |
| **Race** |  |  |  |  |
| Mexican American | 1602 | Ref | 1.25 (0.52, 1.98) | <0.001 |
| Other Hispanic | 1126 | Ref | 1.16 (0.28, 2.05) | 0.010 |
| Non-Hispanic White | 3708 | Ref | 0.33 (−0.09, 0.76) | 0.126 |
| Non-Hispanic Black | 2243 | Ref | 0.80 (0.14, 1.45) | 0.017 |
| Non-Hispanic Asian | 1598 | Ref | −0.05 (−0.81, 0.71) | 0.898 |
| Other Race | 439 | Ref | 0.86 (−0.46, 2.17) | 0.202 |
| **Education level** |  |  |  |  |
| Less than 9th grade | 680 | Ref | 1.28 (0.15, 2.42) | 0.028 |
| 9-11th grade | 1282 | Ref | 1.18 (0.39, 1.97) | 0.004 |
| High school graduate | 2335 | Ref | 0.78 (0.20, 1.37) | 0.008 |
| Some college or AA degree | 3488 | Ref | 0.18 (−0.27, 0.63) | 0.442 |
| College graduate or above | 2929 | Ref | 0.79 (0.29, 1.29) | 0.002 |
| Missing | 2 | Ref | - | - |
| **Marital status** |  |  |  |  |
| Married | 5231 | Ref | 0.40 (0.05, 0.75) | 0.026 |
| Widowed | 145 | Ref | 1.02 (−1.43, 3.46) | 0.417 |
| Divorced | 961 | Ref | 0.75 (0.00, 1.50) | 0.049 |
| Separated | 381 | Ref | 0.65 (−0.76, 2.06) | 0.367 |
| Never married | 2793 | Ref | 1.36 (0.75, 1.97) | < 0.001 |
| Living with partner | 1204 | Ref | 0.44 (−0.42, 1.29) | 0.318 |
| Missing | 1 | Ref | - | - |
| **Poverty income ratio** |  |  |  |  |
| ≤ 1.3 | 3211 | Ref | 0.64 (0.11, 1.16) | 0.017 |
| > 1.3 and ≤ 3.5 | 3532 | Ref | 0.66 (0.19, 1.13) | 0.006 |
| > 3.5 | 3115 | Ref | 0.58 (0.13, 1.04) | 0.012 |
| Missing | 858 | Ref | - | - |
| **Alcohol** |  |  |  |  |
| No | 3279 | Ref | 1.40 (0.95, 1.86) | < 0.001 |
| Yes | 4292 | Ref | 0.12 (−0.31, 0.54) | 0.596 |
| Missing | 3145 | Ref | - | - |
| **Smoking** |  |  |  |  |
| Non-users | 6517 | Ref | 0.79 (0.43, 1.15) | < 0.001 |
| Current smoking | 2415 | Ref | 0.33 (−0.22, 0.88) | 0.235 |
| Past smoking | 1777 | Ref | 0.55 (−0.01, 1.12) | 0.056 |
| Missing | 7 | Ref | - | - |
| **Hypertension** |  |  |  |  |
| No | 7715 | Ref | 0.47 (0.13, 0.80) | 0.006 |
| Yes | 2994 | Ref | 0.94 (0.52, 1.36) | < 0.001 |
| Missing | 7 | Ref | - | - |
| **Diabetes** |  |  |  |  |
| No | 9538 | Ref | 0.52 (0.24, 0.81) | < 0.001 |
| Yes | 1034 | Ref | 1.38 (0.72, 2.04) | < 0.001 |
| Borderline | 144 | Ref | 0.26 (−2.02, 2.54) | 0.823 |
| **Depression** |  |  |  |  |
| No | 9058 | Ref | 0.59 (0.30, 0.88) | < 0.001 |
| Yes | 955 | Ref | 0.98 (0.16, 1.81) | 0.020 |
| Missing | 703 | Ref | - | - |
| **Stroke** |  |  |  |  |
| No | 10558 | Ref | 0.60 (0.33, 0.86) | < 0.001 |
| Yes | 154 | Ref | 0.18 (−2.17, 2.53) | 0.883 |
| Missing | 4 | Ref | - | - |
| **Thyroid disease** |  |  |  |  |
| No | 9980 | Ref | 0.71 (0.43, 0.98) | < 0.001 |
| Yes | 715 | Ref | −0.28 (−1.18, 0.62) | 0.543 |
| Missing | 21 | Ref | - | - |
| **Cancer or malignancy** |  |  |  |  |
| No | 10310 | Ref | 0.62 (0.35, 0.89) | < 0.001 |
| Yes | 404 | Ref | 0.71 (−0.45, 1.88) | 0.231 |
| Missing | 2 | Ref | - | - |
| **Physical activity (MET-h)** |  |  |  |  |
| 0.7−4 | 2089 | Ref | 0.10 (−0.47, 0.67) | 0.720 |
| 4.1−10.7 | 1800 | Ref | 0.12 (−0.53, 0.77) | 0.713 |
| 10.8−24 | 2282 | Ref | 0.85 (0.28, 1.42) | 0.003 |
| 24.1−172 | 2476 | Ref | 0.98 (0.37, 1.59) | 0.002 |
| Missing | 2069 | Ref | - | - |
| **Sedentary time (h)** |  |  |  |  |
| 0−4 | 4006 | Ref | 0.46 (−0.01, 0.93) | 0.057 |
| 4.1−6 | 2220 | Ref | 1.26 (0.67, 1.85) | < 0.001 |
| 6.1−8 | 2035 | Ref | 0.05 (−0.54, 0.63) | 0.876 |
| 8.1−22 | 2411 | Ref | 0.66 (0.15, 1.18) | 0.011 |
| Missing | 44 | Ref | - | - |
| **Energy intake (kcal/d)** |  |  |  |  |
| 14−1545 | 2542 | Ref | 0.87 (0.33, 1.40) | 0.002 |
| 1545.1−2010 | 2542 | Ref | 0.34 (−0.20, 0.88) | 0.220 |
| 2010.1−2603 | 2541 | Ref | 0.77 (0.24, 1.30) | 0.005 |
| 2603.1−9595 | 2544 | Ref | 0.38 (−0.18, 0.94) | 0.180 |
| Missing | 547 | Ref | - | - |

The analysis was adjusted by age, gender, race, education level, marital status, poverty income ratio, physical activity, sedentariness, alcohol, smoking, energy intake, hypertension, diabetes, stroke, depression, thyroid disease, cancer or malignancy. n, number; CI, confidence interval; Ref, reference; d, day.

**Supplementary Table 16.** Examination of the interaction between sleep duration and sleep disorder on body fat percentage.

| **Sleep duration** | **Sleep disorder** | **Model 1^a^** | **Model 2^b^** | **Model 3^c^** |
| --- | --- | --- | --- | --- |
| 7−9 h | No | Ref | Ref | Ref |
| < 7 h | No | −0.53 (−0.94, −0.12) 0.011 | 0.19 (−0.11, 0.49) 0.208 | 0.09 (−0.19, 0.38) 0.518 |
| > 9 h | No | 0.60 (−0.30, 1.50) 0.191 | 0.45 (−0.19, 1.10) 0.171 | 0.12 (−0.50, 0.74) 0.700 |
| 7−9 h | Yes | 2.65 (2.19, 3.12)  < 0.001 | 1.37 (1.03, 1.71)  < 0.001 | 0.75 (0.42, 1.09)  < 0.001 |
| < 7 h | Yes | 1.73 (1.20, 2.27)  < 0.001 | 1.38 (0.99, 1.77)  < 0.001 | 0.64 (0.25, 1.02) 0.001 |
| > 9 h | Yes | 2.01 (0.68, 3.35) 0.003 | 0.93 (−0.02, 1.89) 0.055 | −0.00 (−0.93, 0.92) 0.992 |
| P for interaction | | 0.253 | 0.307 | 0.268 |

^a^ Model 1 adjust for: none. ^b^ Model 2 adjust for: age, gender, race. ^c^ Model 3 adjust for: Model 2 plus education level, marital status, poverty income ratio, physical activity, sedentariness, alcohol, smoking, energy intake, hypertension, diabetes, stroke, depression, thyroid disease, cancer or malignancy. h, hour; Ref, reference.
